# Estrogen Receptors-Mediated Apoptosis in Hormone-Dependent Cancers

**DOI:** 10.3390/ijms23031242

**Published:** 2022-01-22

**Authors:** Adele Chimento, Arianna De Luca, Paola Avena, Francesca De Amicis, Ivan Casaburi, Rosa Sirianni, Vincenzo Pezzi

**Affiliations:** Department of Pharmacy, Health and Nutritional Sciences, University of Calabria, Via Pietro Bucci, Arcavacata di Rende, 87036 Cosenza, Italy; ariannadl@hotmail.it (A.D.L.); paola.avena@unical.it (P.A.); francesca.deamicis@unical.it (F.D.A.); ivan.casaburi@unical.it (I.C.); rosa.sirianni@unical.it (R.S.)

**Keywords:** estrogens, estrogen receptors, apoptosis, intrinsic/extrinsic apoptotic pathways, cancer cells

## Abstract

It is known that estrogen stimulates growth and inhibits apoptosis through estrogen receptor(ER)-mediated mechanisms in many cancer cell types. Interestingly, there is strong evidence that estrogens can also induce apoptosis, activating different ER isoforms in cancer cells. It has been observed that E2/ERα complex activates multiple pathways involved in both cell cycle progression and apoptotic cascade prevention, while E2/ERβ complex in many cases directs the cells to apoptosis. However, the exact mechanism of estrogen-induced tumor regression is not completely known. Nevertheless, ERs expression levels of specific splice variants and their cellular localization differentially affect outcome of estrogen-dependent tumors. The goal of this review is to provide a general overview of current knowledge on ERs-mediated apoptosis that occurs in main hormone dependent-cancers. Understanding the molecular mechanisms underlying the induction of ER-mediated cell death will be useful for the development of specific ligands capable of triggering apoptosis to counteract estrogen-dependent tumor growth.

## 1. Introduction

Apoptosis is a programmed physiological mechanism of cell death. It is a genetically controlled process that plays a critical role in embryonic development [1], tissue regeneration [2], elimination of genome-damaging cells [3], and cancer prevention [4]. In the adult organism, it contributes—together with mitosis—to the cellular numerical homeostasis maintenance [5]. Apoptosis involves both distinct morphological characteristics and energy-dependent biochemical changes [6,7]. It causes cell rounding and loss of cell–cell contacts, changes in the membrane phospholipids distribution and potential mitochondrial membrane leading permeability increase, nucleus, and cytoplasm condensation followed by cellular fragmentation into apoptotic bodies [6]. Biochemical modifications that apoptotic cells exhibit include protein cleavage, DNA breakdown, proteolytic caspases activation, and phagocytic recognition by macrophages [7,8].

**Figure 1:**
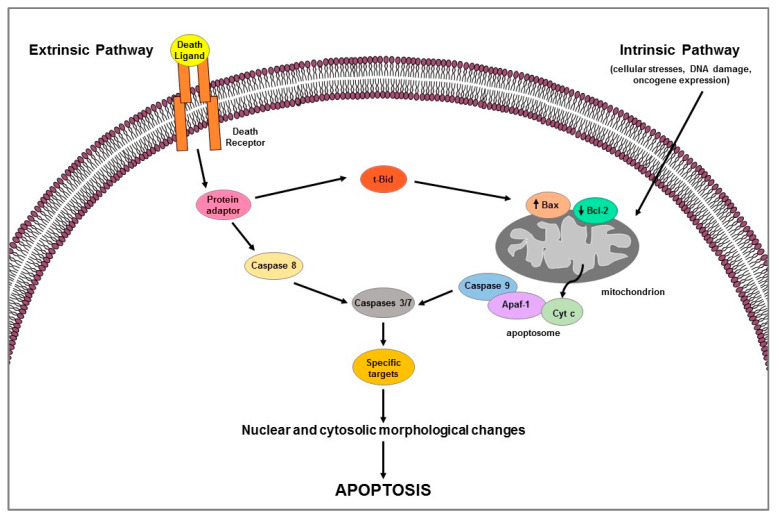
Schematic representation of extrinsic and intrinsic apoptotic pathways.

Apoptosis is a highly complex process that involves different cascades of molecular events. Two main pathways are known: the extrinsic or death receptor pathway and the intrinsic or mitochondrial pathway [8,9] (Figure 1). They are carried out by caspases, a family of cysteine-dependent aspartate-directed proteases that cleave specific target proteins (e.g., Parp-1) [10,11]. Each apoptotic pathway activates its own initiator caspase (e.g., caspase 8 for extrinsic pathway; caspase 9 for intrinsic pathway) which in turn activate the executioner caspase (e.g., 3 or 7) resulting in nuclear and cytosolic morphological changes and finally in cell death [11].

Extrinsic pathway uses extracellular signals or death ligands (e.g., Fas-L, TRAIL, TNF) that, by binding their cognate-death receptors (Fas, TRAIL, and TNF receptors), recruit adaptor proteins (e.g., FADD, TRADD) forming the death-inducing signaling complex (DISC); the latter activates caspase 8 which in turn cleaves and activates the executioner caspases [7] (Figure 1). Death receptor-induced apoptosis can be inhibited by cFLIP which, competing with pro-caspase 8 for binding to FADD, blocks caspase 8 processing and then its activation [12]. Intrinsic apoptotic mechanism, that can be stimulated by several signals such as cellular stresses (i.e., hypoxia, radiation, toxins, growth factor deprivation), DNA damage, or oncogene expression, involves the mitochondria and mitochondrial proteins [7,8] (Figure 1). The overall pathway is regulated by the B-cell lymphoma-2 (BCL-2) protein family which includes members containing BH1-4 domains. Several proteins, grouped according to their function in: (1) anti-apoptotic proteins (Bcl-2, Bcl-xL, Bcl-w, Mcl-1, Bfl-1/A1), (2) pro-apoptotic pore-formers (Bax, Bad, Bak, Bok), and (3) pro-apoptotic BH3-only proteins (Bid, Bik, Bim, Bmf, Hrk, Noxa, Puma, etc.) belong to the Bcl-2 family [13]. Pro-apoptotic members upregulation induces changes in the mitochondrial outer membrane permeability leading to cytochrome c (Cyt c) release into cytosol; free Cyt c binds Apaf-1 and caspase 9 to form the apoptosome complex which subsequently activates caspase-3 and -7 triggering apoptotic cell death [13]. The two apoptotic pathways above described can occur not only separately but can be linked by the activation of some proteins (e.g., tBid) thus affecting each other [13] (Figure 1). Apoptotic process can be regulated by p53, a tumor suppressor that is able to modulate key control points in both intrinsic and extrinsic pathways [14]; it transcriptionally upregulates apoptosis-related proteins (i.e., Puma, Noxa, Bid, and Bax) expression and physically interacts with and neutralizes the anti-apoptotic activity of Bcl-2 and Bcl-xL; moreover, it can transactivate the death receptor genes and/or induce those (e.g., PTEN) that inhibit antiapoptotic pathway such as the survival PI3K/AKT signaling [14].

The apoptosis evasion is a cancer cell hallmark, beyond uncontrolled growth and angiogenesis. Tumor cells by losing apoptotic control survive longer, accumulating mutations over time that can increase invasiveness, stimulate angiogenesis, deregulate cell proliferation, or interfere with differentiation [15]. Cancer cells can modulate apoptotic pathways at transcriptional, translational, and post-translational level. They escape apoptosis by: (1) increasing or decreasing expression of anti- or pro-apoptotic genes, respectively; (2) changing anti- or pro-apoptotic proteins functions through post-translational modifications, such as phosphorylation; or (3) losing caspases function [16]. Therefore, a promising antitumor therapeutic approaches may consist in the restoration of one or both apoptotic pathways through antiapoptotic factors inhibition and/or proapoptotic molecules stimulation [17]. The strategies aimed at making tumors responsive to death receptor-induced apoptosis include the downregulation of a master anti-apoptotic protein c-Flip by metabolic inhibitors [18] and the caspase 8 activation by interferon [19]. Another approach to induce apoptosis by extrinsic pathway involves the use of antibodies with agonistic activity against TRAIL death receptors (DR4 and DR5) or TRAIL soluble recombinant derivatives (sTRAIL) [20]. Intrinsic pathway mediators are also targeted for anticancer therapeutic approaches. Pharmacological inhibitors of anti-apoptotic proteins typically overexpressed in tumor cells, such as Bcl-2 and Bcl-xL, transcriptional inhibitors of the Bcl-2 gene, or small molecules that reactivates the wild-type function of mutant p53, have been developed [21].

It is known that estrogens can support cell survival or induce cell death by apoptosis depending on the ERs subtype present in cells [22]. Cellular levels of ERα and ERβ are reported to be important determinants of response to E2 and selective estrogen receptor modulators [23]. Although these receptors share a structural homology, they produce different effects and their unbalanced expression could play a pivotal role in the development and progression of tumors [24]. A progressive loss of ERβ expression during the process of carcinogenesis has been documented in prostate [25] and breast [26] cancers, suggesting a role for ERβ as a potential inhibitor of cellular proliferation and/or transformation [27]. Data from cell cultures and gene expression suggest that E2-activated ERβ, by antagonizing ERα activity on E2-responsive promoters [28], may act as a tumor suppressor, thus modulating ERα proliferative effects [29,30,31]. E2/ERα complex can either bind directly to DNA (classical pathway) or indirectly via protein–protein interactions (nonclassical pathway) and regulate the transcription of factors playing an important role in proliferation, differentiation, survival, and angiogenesis [32]. It has been also reported that E2/ERα mediates proliferative effects through rapid non-genomic mechanisms originating at cell membrane level [33]. In fact, the membrane E2/ERα complex can rapidly activate multiple signal transduction pathways (i.e., ERK/MAPK, PI3K/AKT) involved in both cell cycle progression and apoptotic cascade prevention [34]. Non-genomic actions have been also reported for ERβ; membrane E2/ERβ complex can drive the rapid and persistent phosphorylation of p38/MAPK which, in turn, is involved in caspase 3 activation and Parp-1 cleavage, leading to apoptosis [31,34]. Therefore, the opposite effects elicited by E2 in cells where both ERα and ERβ are expressed may depend on the balance between signals originating from each isoform [35]. Furthermore, several studies revealed that estrogens act also through GPER, a member of GPCR cell-membrane proteins superfamily [36,37]. GPER can mediate rapid E2-induced non-genomic signaling events, including activation of MAPK which can induce both proliferative pathways as well as apoptotic events [38,39,40].

In this review, we summarized the current knowledge on the involvement of classical ERs and its splice variants on apoptotic mechanisms that occurs in hormone-dependent cancers such as breast, prostate, ovarian, and endometrial. Moreover, the role ERs and that of GPER in the apoptosis regulation in testicular and adrenocortical cancers are also discussed.

## 2. Duality of Estrogen Receptors Function in Cancer

**Figure 2:**
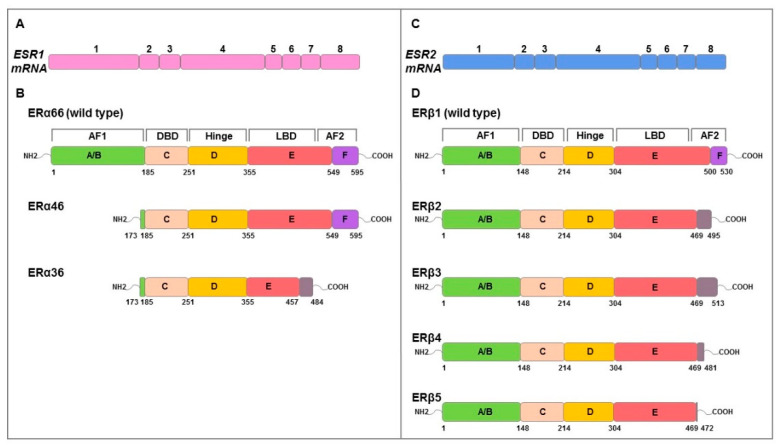
Schematic representation of genomic and functional structure of ERs. The numbered boxes illustrate the eight exons of *ESR1* (pink) (**A**) and *ESR2* (blue) (**C**) mRNAs that encode ERα (**B**) and ERβ (**D**) proteins, respectively. Both structural domains (A–F) and functional domains (AF1, hinge, DBD, LBD, AF2) are indicated. The amino acids numbers of structural domains are indicated in black below. Full length ERα and ERβ is 595 and 530 amino acids in length, respectively. ERα46 and 36 lacks exon 1 resulting in a truncated form of receptor that is missing the first 173 amino acids of the full-length sequence. ERα36 also lacks the last 138 amino acids encoded by exons 7 and 8 which are replaced by 27 amino acids at the C terminus. ERβ isoforms (ERβ2, 3, 4, 5) isoforms differ at common point in the peptide sequence (amino acid 469) and result of alternative splicing of exon 8. Grey boxes represent the alternative specific amino acid sequences for each isoform. Adapted from Gibson et al. [43].

E2 effects are mediated by ERα and ERβ which are coded by *ESR1* and *ESR2* genes, respectively. They have a molecular structure of six protein domains, denoted as A to F, encoded by eight exons, which differ in their functions: domain with ligand-independent activation function (AF1) at the N-terminus (A/B domain), DNA-binding domain (DBD, C domain), hinge domain (D domain) containing nuclear localization signals, E/F domains including ligand-binding domain (LBD), and the activation function 2 (AF2) with hormone-dependent activity and agonist/antagonist regulator sites [41,42,43] (Figure 2). ERs possess a similar structure and share considerable homology in both DBD and LBD [41]. Alternative mRNA splicing mechanisms differentially regulate ERs isoforms expression producing different variants (ERα66/46/36; ERβ1/2/3/4/5) (Figure 2) with specific ligand binding, subcellular localization, response to post-translational modification and both ligand-dependent and -independent functions [41,43].

ERα consists of 595 amino acids with a molecular weight of 66 kDa (ERα66 wild type). In recent years, two other shorter isoforms, ERα46 and ERα36, have been characterized. The 46 kDa isoform lacking the N-terminal A/B or AF1, is expressed in various cell types, such as macrophages [44], vascular endothelial cells [45], osteoblasts [46], and also in cancer cells [42,47]; the 36 kDa isoform differs from classical ERα66 due to the lack of both AF1 and AF2 transcriptional activation domains while retaining the DNA binding domain, the partial dimerization and the ligand-binding domain [47]. ERα36 is characterized by a single domain of 27 amino acids instead of the last 138 amino acids encoded by both ERα46 and ERα66 gene; this domain is responsible of ligand binding domain alteration on which depends ERα36 different binding affinity [42].

ERα66 is found mainly in the cytoplasm and in the nucleus but with some surface localization, ERα46 is distributed similarly to ERα66, while ERα36 resides primarily in the plasma membrane but can be found in both cytoplasm and nucleus [42,47]. In humans, the ERβ isoform of 530 amino acids (ERβ1 wild type) and the shorter isoforms of 495 (ERβ2), 513 (ERβ3), 481 (ERβ4), and 472 (ERβ5) amino acids, resulting from alternative splicing of exon 8, are known [48]. At the subcellular level, these isoforms can be localized in the nucleus, cytoplasm, and at the cell membrane [34,49]. Among the human ERβ isoforms, ERβ1 is the only one that has been shown to have full function [50]. It has been highlighted that both ERα and ERβ1 require binding with the ligand and ERβ1 can form both homodimers and ERα/ERβ1 heterodimers [51,52]. However, ERβ2 is able to form heterodimers with ERα or ERβ1 without ligand [52] inhibiting the binding to ERE sequences of both receptors; this suggests how ERβ2 acts as a functional modulator of ERα and ERβ1.

Although both full-length receptors bind endogenous ligands (i.e., E2) with comparable binding affinities, their affinity for various natural and synthetic ligands—including phytoestrogens and pharmaceuticals—differs significantly [53,54,55,56,57]. ERs regulate complex and dynamic gene expression networks involved in the regulation of both tumor cell proliferation and death. The expression pattern of the two ERs isoforms in tissues is species-specific and also cell-specific [58,59,60,61].

Estrogen/ERs complex can trigger both genomic and non-genomic signaling [62,63,64,65]. In the genomic pathway, this complex binding ERE, either directly or indirectly via transcription factors, modulates gene expression in several tissues [65,66]. In addition to ligand-dependent activity of ERs, ligand-independent pathways mediated by ERs have also been described [65]. ER-mediated transcription is induced by kinases whose activation depends on growth factor signaling. This molecular mechanism can explain the hormone-independent growth of some tumors [67]. It has been reported that estrogen-activated ERα66 dimers result in ERE-driven transcriptional proliferative responses while ERα46 has a repressive role in ERE-mediated transcription, either through interference with ERα66 binding or through recruitment of transcriptional repressors [42]. A negative regulatory role in estrogen genomic signaling is exerted by ERβ through a transcription inhibition of proliferation-related genes and/or transcription activation of apoptosis-related genes [42]. ERα36 dimers do not directly drive any transcriptional activity but primarily mediate estrogen rapid effects [68]. The rapid estrogen-mediated effects, named non-genomic activity [62,65,69], involve the generation of the second messengers Ca^2+^, cAMP, NO, as well as receptor tyrosine kinases activation—such as EGFR, IGF1R, and protein/lipid kinases (e.g., PI3K, AKT, MAPK, SFKs, PKA and PKC) [70,71,72,73]. The ERs non-genomic functions requires S-palmitoylation which allows ERα and ERβ localization at the plasma membrane, where they associate with caveolin-1 [34]. After E2 stimulation, ERα dissociates from caveolin-1 and activates rapid signals leading to cell proliferation increase. By contrast, E2 increases ERβ association with caveolin-1 by activating p38 kinase and the downstream pro-apoptotic cascade (i.e., caspase 3 and Parp-1 activation) [34].

Non-genomic estrogens responses can be mediated by GPER in both normal and cancer cells [37,74]. Particularly, GPER activation by E2 and/or specific ligand of GPER, G1 [75], determines multiple intracellular events such as EGFR transactivation leading to rapid ERK1/2 activation, PLC and PI3K phosphorylation, AC stimulation, and intracellular calcium mobilization [37,69,76,77,78] involved in cell proliferation and apoptosis modulation [38,39,40,79]. GPER involvement in breast [80,81], endometrial [82], and ovarian [83] cancer progression has been reported. However, studies demonstrated that it can mediate anti-proliferative effects also in BC [79], PC [84], and OC [85] and can induce apoptosis in LCT [38] and ACC [40] cell lines.

While the classical ERs activities in the etiology and progression of many hormone-responsive tumors are well defined, the specific role of each receptor and their expressed splice variants in estrogen-responsive tumors remains unclear. Knowledge about the complex interaction between differentially expressed nuclear ERs has been extended by recent advances on different splice variants’ characterization and the availability of new experimental cancer models.

ERα is associated with poor prognosis and malignancy in breast, prostate, ovarian, and endometrial cancer by modulating both tumor onset and progression [86,87,88,89,90,91]. The involvement of ERα in both BC and PC progression has been confirmed by functional studies on ERα knockout mice [92,93]. Emerging evidence indicates that ERα is crucial for PC progression by acting as an oncogene [90], inducing EMT [94] and MPs activation [95]. In BC, ERα through a crosstalk with IGF1R signaling pathway enhances cancer growth [96]. In ER + breast cancer cells E2 through ERα/PI3K/AKT/mTOR [97] and Ca^2+^-mediated [98] signaling pathways activation induce tumor progression. Furthermore, in BC cells, estrogen/ERα complex stimulates downstream signaling pathways leading to EMT and ECM remodeling [99,100].

An involvement in tumor growth and progression, metastatic potential, resistance to drug treatments, and poor prognosis has been confirmed for ERα36 [101,102,103]. High levels of expression of this splice variant have been found in several cancer types such as gastric, colorectal, renal, lung, thyroid, laryngeal, endometrial, hepatocellular, neuronal, and breast cancers [101,103]. In Ishikawa EC cells, extra-nuclear ERα36 mediates the non-genomic estrogen/PKCδ/ERK signaling pathway activation, which increases CD1/CDK4 expression and therefore tumor growth [104]. In BC, the E2-induced ERα36 rapidly recruits Src at plasma membrane and stimulates downstream cascades, including MEK1/ERK activation and PXN phosphorylation resulting in CD1 expression increase and then in cell proliferation increase [105]. Moreover, in the same tumor, membrane E2/ERα36 complex can initiate a PKCδ/ERK signaling cascade which affect cell proliferation as well as phosphorylation of transcription factors that activate metastasis and tumor aggressiveness [106]. It has been reported that ERα36 forms heterodimers with ERα66 or ERβ, affecting the transcription activities of both ERα66 and ERβ. Furthermore, ERα36 may retain ERα66 in the cell cytoplasm to block its genomic signaling [107]. However, ERα36 expression is subjected to negative regulation by ERα66 [108]. This suggests that the relative expression levels of ERα66 and 36 in a specific cell determine ratios of genomic or non-genomic estrogen signaling. In fact, the reduction in ERα66 protein expression levels, that correlates with ERα36 increase, represents one of the mechanisms underlying to antiestrogenic therapy resistance [107]. It has been reported that cells expressing high levels of ERα36 are more sensitive to E2, induce the MAPK/ERK signaling pathway at lower E2 concentrations and are responsible for the escape of the estrogens genomic signal [109]. Similarly, ER-negative BC MDA-MB-231 and MDA-MB-436 cells that express high levels of endogenous ERα36 show that low doses of tamoxifen induce MAPK/ERK pathway activation, while high doses fail to do the same. This could depend by different concentrations of antiestrogens that determine changes in ERα36 conformations and/or its functions [110].

ERβ estrogen-mediated stimulation has been reported to counteract the growth of estrogen-responsive tumors such as breast and prostate [27,111]. Overall, the ERβ function is thought to be antiproliferative and proapoptotic, therefore, it is considered as a tumor suppressor [111,112]. The E2/ERβ complex does not activate any of the signal molecules activated by E2/ERα involved in cell growth modulation [113], but instead drives apoptotic cell death [31]. It has been suggested that ERβ may antagonize ERα function through heterodimerization with ERα leading to a decrease in the estrogens transcriptional activity and proliferative effects in BC cells [52,114]. Studies reported that the pure anti-estrogen fulvestrant increased ERβ expression at both mRNA and protein levels in ERα+/ERβ+ as well as in ERα-/ERβ + BCs [115]. In particular, in MCF-7 (ERα+/ERβ+) BC cells, it synergized with TAM in inhibiting cell proliferation and inducing apoptosis. In TNBC cell models MDA-MB-231 (ERα-, ERβ high) and MDA-MD-468 (ERα-, low ERβ) cells, fulvestrant, by upregulating ERβ, also reduced cell growth [115]. However, other reports showed that ERβ activation increased cell proliferation in TNBC [48]. ERβ stimulation in ERα- BCSCs increased mammospheres formation while the use of PHTPP, a selective ERβ antagonist, reduced it [116]. In the same way, ERβ knockdown inhibited the growth of murine TNBC xenograft models [116].

In BC, ERβ function also depends on the expression of several splice variants. A meta-analysis study performed in BC patients (ER + or −) showed that ERβ1 is positively associated with improved overall and disease-free survival and predicted response to endocrine therapy [117]. However, in the presence of ERα, the positive association of ERβ1 with overall survival was lower, suggesting that this event also depends on the co-expression of ERα [117]. In another work, it has been demonstrated that ERβ2 activation induced proliferation and invasiveness in TNBC cells [118]. Using immunohistochemistry analysis in a large cohort of BCs with long-term follow-up, the prognostic significance of ERβ1, ERβ2, and ERβ5 has been evaluated. In ERα + BC, nuclear expression of ERβ2 was associated with better clinical outcome, while cytoplasmic ERβ2 expression correlated with poor survival; nuclear ERβ5, but not ERβ1, significantly was related with better overall survival [49]. These data suggest that specific splice variants, and also their cellular localization, differentially affect outcome.

A much-reduced expression of ERβ was found in the advanced stages of PC [25,27]. In this tumor, while ERα is associated with aberrant proliferation and malignant development, ERβ expression correlates with cell proliferation inhibition, differentiation, and apoptosis [119]. An ERβ suppressor role has been confirmed by ERβ agonists use that was able to reduce tumor growth in both in vitro PC cell lines and in vivo xenograft murine models [120]. It has been shown that the ERβ activation upregulates tumor suppressor genes while its loss increases AR signaling in PC [121]. Moreover, while high ERβ1 expression is associated with better overall survival, high ERβ2 expression is correlated with poor survival in PC [122]. The negative prognostic role for nuclear ERβ2 was demonstrated in a study performed on 144 specimens of PC [123]. Additionally, the co-expression of nuclear ERβ2 and cytoplasmic ERβ5 was associated with a poor prognosis in terms of survival time [123]. These results suggest that both ERβ2 and ERβ5 may promote tumor prostate progression, although further studies are needed to elucidate the differential roles of ERβ and its splice variants in this cancer type.

## 3. ERs-Mediated Apoptosis in Hormone-Dependent Cancers

Several studies show that classical ERs can be involved in inducing apoptosis in some hormone-dependent tumors through the involvement of both extrinsic and intrinsic apoptotic pathways [124,125,126,127,128]. In order to establish the specific role of the various ERs in apoptosis regulation of hormone-dependent cancers, the co-expression of isoforms, the splicing variants expression, and their intracellular localization must be carefully evaluated. In the following subsections, we will summarize the results obtained on this topic in breast, prostate, ovarian, and endometrial cancers.

### 3.1. Breast Cancer

BC, the most common tumor among women worldwide, is recognized as the most representative hormone-dependent disease [129]. On the basis of specific receptors presence or absence, it can be grouped into four distinct molecular subtypes: (1) luminal A (ER/PR positive, HER2 negative), (2) luminal B (ER and/or PR positive, HER2 positive), (3) HER2 over-expressing (HER2 positive alone), and (4) basal-like triple negative (negative for all three receptors) [130].

BRCA1 and BRCA2, known as regulators of DNA repair, transcription, and cell cycle in response to DNA damage, are the most commonly mutated genes and associated with a high risk of BC [131]. Furthermore, other mutations or inactivation of some genes such as PTEN, STK11, CDH1, ATM, P53 [131] or amplification of others such as CD1 occur in this cancer [132].

ERs are not only involved in the development of BC but play a vital role in its prognosis and occurrence. It has been reported that one of the risk factors for the initiation and progression of BC is the increase in endogenous and exogenous estrogens levels [133]; however, the expression of specific nuclear ER isoforms influences tumor response to hormone [134].

Studies revealed how in the mammary gland ERα acts as primary player: a severe impairment of its development is observed in ERα knockout mice [135], while ERβ ablation shows less impact [136]. About 70% of human BCs express ERα, and the majority of ERα-positive BCs also express ERβ [137]. However, the lack of selective ligands and poor antibody quality prevented the ERβ role in BC from being well-elucidated. It is reported that ERα mediates proliferative effects in BC [138], while differentiative, antiproliferative, and proapoptotic roles are attributed to ERβ [30,114], even if a proliferative and pro-survival property are also reported [139]. In addition, there is evidence for a role not only of ER but also of PR in driving BC; PR exerts paradoxical effects on BC growth depending on the experimental conditions and on different activity of both PR-A and PR-B isoforms [140,141].

Treatment of hormone receptor positive BCs is mainly based on endocrine therapy and chemotherapy [142]. Although estrogens are commonly reported to stimulate the growth of BC, it can be used as an effective treatment for this disease (the “estrogen paradox”) [143,144]. In fact, studies indicate that high-doses estrogens are effective for the treatment of advanced BC, both as a first-line treatment and for treatment after the onset of endocrine resistance to antiestrogens (e.g., TAM) and AI [145]. However, a long period of estrogen deprivation is required before starting therapy for treatment to be effective (the “gap hypothesis”) [146]. It has been suggested that BC cells can adapt to low levels of estrogens by enhancing their sensitivity to E2 [147]. Generally, in ER + BC cells with estrogen deficient environment, E2 hypersensitivity correlates with apoptosis induction [148]. In particular, higher doses of E2 increase both ER expression and non-genomic and genomic estrogen signaling which allow apoptotic death of tumor cells [148]. Paradoxically, antiestrogen treatment may also provoke compensatory ER overexpression and estrogen synthesis in tumor cells, restoring the apoptotic capacity of estrogen signaling that results in transitory tumor regression. Subsequently, the loss of the ability of antiestrogens to increase estrogenic signaling can lead to uncontrolled proliferation and acquired antiestrogen resistance. However, high doses of estrogens are able to restore the suppressed estrogen signaling even after prolonged treatment with antiestrogen. In this context, both exogenous and newly synthesized estrogens compete with antiestrogen for binding to newly expressed ERs; so the estrogenic signaling predominance leads to apoptotic cell death and regression of the disease [148].

**Figure 3:**
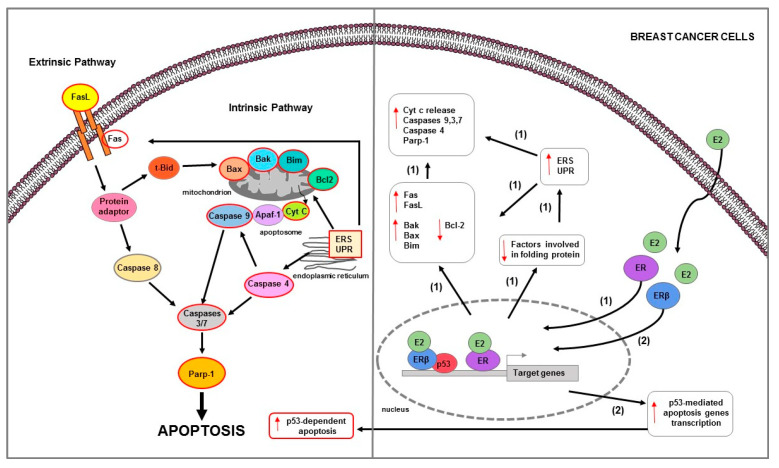
Main mechanisms of estrogen-induced apoptosis in breast cancer cells. On the right side of the figure, the main transcriptional mechanisms mediated by ERs are indicated; on the left side, are illustrated apoptotic pathways (highlighted by the red outline) consequently activated. Both extrinsic and intrinsic pathways are involved in apoptosis of long-term estrogen deprived BC cells (1). Additionally, in BC MCF-7 cells (2), ERβ interacts with p53 and abrogates both the ERα-p53 binding and ERα-dependent suppression of p53-mediated apoptosis genes transcription. The red arrows pointing up indicate activation and/or expression increase, while those pointing down indicate inhibition and/or expression decrease. See text for more details.

These phenomena have been observed in several in vitro studies that used experimental models of variants of BC MCF-7 cells developed from either long-term estrogen deprivation (LTED [147], E8CASS cells [149], MCF-7:5C [150]) or long-term exposure to selective ER modulators (SERM, TAM, or RLX [151,152,153,154]) which responded paradoxically to E2 with apoptosis. In these cells, apoptotic mechanisms involve both membrane death receptors as well as mitochondria-mediated pathways [124,155] (Table 1) (Figure 3).

Osipo et al., demonstrated that in mouse xenograft models using MCF7 stimulated with long-term tamoxifen (MCF-7TAMLT), E2 caused tumor regression by inducing Fas receptor and suppressing the antiapoptotic/prosurvival factors NF-κB and HER2/neu [154]. Similary, E2 apoptotic actions were observed in raloxifene-resistant MCF-7 cells (MCF-7RLX) [153]. Recently, response to E2 treatment and anti-estrogen withdrawal was assessed in fulvestrant-resistant MCF-7 (MCF7FR) and LTED cells. E2 treatment and fulvestrant withdrawal induced transcriptional activation of ER and thus made adapted cells hypersensitive to estrogen [156]. Specifically, estrogen-induced cell death was facilitated by UPR activation mediated by IRE1a which then downstream driven p53 and JNK signaling and subsequent apoptosis. Apoptotic effects of E2 and fulvestrant withdrawal were confirmed by Bim protein expression increase and Parp-1 cleavage [156]. Estrogen-mediated apoptosis was demonstrated in LTED cells where estradiol activated Fas/FasL signaling pathway that induced caspase activation and DNA fragmentation [146]. Similarly, in both LTED and E8CASS cells, E2 significantly inhibited cell growth primarily through a pro-apoptotic action involving caspase 9 and 7 activation and NF-kB levels decrease [157]. Additionally, in these cells, the silencing of mitochondrial protein Bcl-2 that was upregulated synergistically enhanced the estrogen pro-apoptotic effect and concomitantly decreased cancer cell growth [157]. Another study performed in E8CASS cells evidenced that the zinc finger protein E9—a transcriptional factor involved in signal transduction, phosphorylation, and nucleotide transport—represented the mediator for estrogen-induced apoptotic response in BC [158]. In these cells, the E9 mRNA levels increase correlated with estrogen-induced cell proliferation inhibition and genomic DNA degradation [158]. Molecular mechanisms of estrogen-mediated apoptotic cell death were also evaluated in MCF-7:5C. In this cell model, E2 treatment caused mitochondrial transmembrane potential decrease, Cyt c release, Bax, Bak, Bim, and P53 protein expression increase together with caspase 9, caspase 7, and Parp-1 cleavage. In addition, siRNA targeting of Bax, Bim, and P53 dramatically reduced the ability of E2 to induce apoptosis in these cells [159]. In another study, it has been observed that MCF-7:5C cells responded to E2 by suppressing ERα signaling and producing ERS and inflammatory response [160]. Estrogen signaling was suppressed by upregulating genes that reduce intracellular E2 concentrations or that antagonize ERα activity and by repressing genes that promote ERα activity [160]. Moreover, in the same study, it has been revealed the inhibition of genes is involved in protein folding and in the degradation of misfolded proteins, with consequent accumulation of unfolded/misfolded proteins [160]. These molecular events can result in UPR activation which inhibits the translation of proteins to relieve stress and activated that of proapoptotic BCL-2 family members. It is known that the UPR includes three primarily cytoprotective adaptive pathways that are highly coordinated and act to attenuate the protein load using three sensors: IRE1-α, ATF6, and PERK [164]. However, in the absence of protein synthesis homeostasis restoration following prolonged ERS, this system can lead to cell death by apoptosis [165]. In fact, in MCF-7:5C cells, E2-dependent apoptosis after ERS was confirmed by Bim and Bax expression increase, Parp-1 cleavage, and activation of caspase 4 which is known to induce downstream caspases cleavage and cause apoptosis [160]. In addition, the E2-mediated upregulation of proinflammatory genes IL, IFN, and arachidonic acid-related genes contributed to induce apoptosis in a synergic manner [160]. Lui et al. demonstrated that in MCF-7:5C, targeting IFITM1, a critical downstream protein of IFNα signaling pathway, caused apoptotic cell death [161]. Specifically, IFITM1 suppression decreased cell proliferation and invasion by JAK/STAT-mediated p21 increase and promoted cell death as evidenced by Parp-1 cleavage and DNA fragmentation [161]. Moreover, the loss of MUC1, a key regulator of IFITM1 transcription, alone and in combination with E2 treatment inhibited cell proliferation and induced apoptosis as confirmed by TUNEL-positive MCF-7:5C cells [126]. In the same cell model, estrogen-induced ERα/cSrc interaction was followed by an increase in ROS and HO-1 expression levels, leading to ERS and mitochondrial stress [162]. These events triggered UPR and intrinsic and extrinsic apoptotic pathways activation [162]. It is known that the therapeutic target for preventing stress responses in cancer is NF-κB. NF-κB constitutive activation is one of the stress responses required for adaptation to long-term E2 deprivation [166]. It has been demonstrated that in MCF-7:5C cells, E2-induced apoptosis occurred through activation of PERK/STAT3/NF-κB/TNFα signaling pathway [163]. In particular, E2 modulated NF-κB activity differently on the basis of treatment time. Initially, nuclear E2/ER preferentially activated C/EBPβ which can suppress NF-κB DNA binding and NF-kB-mediated activation of TNFα. However, after a long period of treatment, E2 increased DNA binding activity of NF-kB p65, leading to TNFα induction and related apoptosis. Specifically, E2 stimulated PERK which in turn phosphorylated STAT3 that played an essential role in the late NF-κB activation and apoptosis [163].

Data confirm that PERK pathway activation increases phosphorylation of eukaryotic eIF2a protein at serine 51 residue leading to global protein synthesis inhibition and endoplasmic reticulum protein load attenuation [164]. However, sustained phospho-eIF2a–mediated translational repression can also initiate cell death through ATF4 and CHOP expression increase and then caspase cleavage [167]. PERK-mediated phosphorylation of eIF2a upregulate GADD34 expression that acts as a negative feedback loop by dephosphorylating eIF2a and promoting recovery from translational inhibition in the UPR [168,169]. Moreover, dephosphorylation of eIF2a is also catalyzed by CReP [170]. Recently, Sengupta et al.—in order to elucidate estrogen-induced apoptosis in MCF7:5C cells—demonstrated that cell death occurred after PERK and eIF2α phosphorylation increase that were responsible of ATF4 and CHOP expression and Parp-1 cleavage increase. Moreover, pharmacologic (salubrinal) and genetic inhibition (siRNA) of GADD34 and CReP mimicked estrogen action by maintaining eIF2α phosphorylation state [127].

Furthermore, in BC cells, estrogen-mediated apoptosis depends on the expression of specific nuclear ER isoforms. It has been observed that in MCF-7 cells (ERα+/+ and p53+/+), the presence of ERβ attenuated ERα-induced cell proliferation, reversed its transcriptional activation and inhibition ability and increased apoptosis [134] (Figure 3). In particular, ERβ physically interacted with p53, abrogated the ERα-p53 binding and thus antagonized the ability of ERα to suppress p53-mediated transcriptional activation of genes involved in both cell proliferation (e.g., p. 21) and apoptosis (e.g., DR5, Bax). ERβ also affected chromatin-modifying enzymes that could alter chromatin accessibility [134]. It abrogated the H3K9me3 repressive chromatin conformation by downregulating SUV39H1 and SUV39H2, and induced H3K4me3-mediated epigenetic activation of ERα-repressed and p53-stimulated gene p21 [134]. Furthermore, ERβ also reduced the ERα-mediated recruitment of N-CoR and SMRT corepressors, attenuating the crosstalk between ERα and p53 [134].

### 3.2. Prostate Cancer

**Figure 4:**
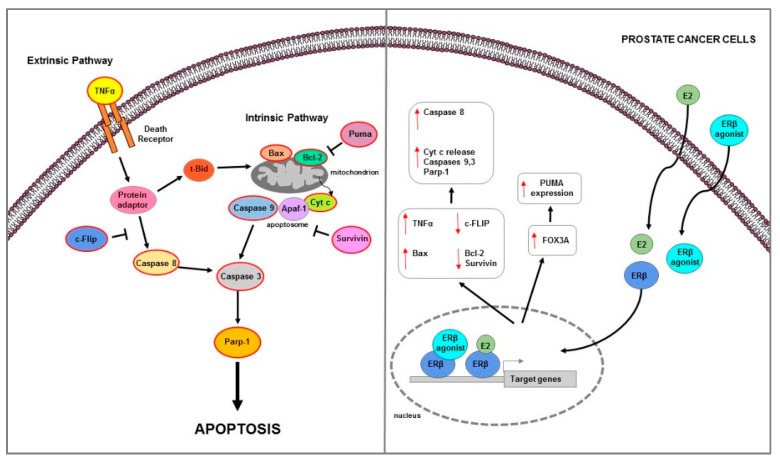
Main mechanisms of estrogen-induced apoptosis in prostate cancer cells. On the right side of the figure, the main transcriptional mechanisms mediated by ERs are indicated; on the left side, is an illustration of how the apoptotic pathway (highlighted by the red outline) is consequently activated. Both extrinsic and intrinsic pathways are involved in apoptosis of PC cells. The red arrows pointing up indicate activation and/or expression increase, while those pointing down indicate inhibition and/or expression decrease. See text for more details.

PC represents the second most common and fifth most aggressive neoplasm among men worldwide [171]. Among the various identified risk factors, the best known include race (African men are at higher risk), genetics (e.g., BRCA1/2 mutations), and obesity [172]. Recently, Cimadamore et al., summarized the main novelties in prognostic and therapeutic markers in PC [173]. The authors evidenced that on the basis of the prognostic and therapeutic tissue markers in PC patients, two groups are considered [173]. The first group, including two subgroups—one involving only the morphological evaluation (i.e., PC degree), and the other involving both morphological and immunohistochemical evaluations (i.e., PC aggressive, AVPC)—is related to prognostic markers based on morphological and immunohistochemical evaluations. The other large group is based on molecular markers (i.e., DNA somatic mutations) that predict severe disease or a response to therapy [173]. The therapeutic strategies for PC treatment are rapidly evolving [174]. Targeting of AR signaling represents one of the main therapeutic options. Currently, surgical and chemical castration, with LHRH analogues and AR signal inhibitor, are used for all forms of advanced disease [175]. Although ADT has been widely prescribed for patients with advanced PC, it gradually acquires a lethal phenotype and results in CRPC during ADT. In addition to AR, ERs may be involved in the development or regulation of PC. SERMs have been developed and an approach has been tested in which the EAB through a combination of toremifene and ADT improves the biochemical recurrence rate in metastatic bone PC [176]. Several findings indicate that estrogens play an important role in growth, differentiation, and homeostasis of normal prostate tissues, as well as in PC pathogenesis. The first clinical evidence that hormones can influence the development of PC have been reported by Huggins and Hodges that indicated how androgens promoted tumor growth and estrogens inhibited it [177]. Estrogens can affect prostate cancer growth reducing FSH production and breaking down hypothalamic pituitary stimulation by LH, which in turn reduced androgen synthesis. Nevertheless, in CRPC, estrogens therapy has not proved effective because cells can overcome the mechanisms mentioned above and progress in the disease [178]. Furthermore, cardiovascular side effects can be caused by estrogenic therapy, which limits its clinical use as an alternative to castration [179]. These undesirable effects of estrogenic drugs are probably partly mediated by the classical ER transactivation route [180]. Studies showing that estrogens through ER activation reduce the progression of different forms of PC are always growing. Prostate tissue expresses besides the AR [181], also expresses ERs [112]. ERα and ERβ are differently localized: ERα is mainly expressed in stromal cells within the non-malignant human prostate, occasionally in basal-epithelial cells, whereas ERβ is mainly detected in basal-epithelial cells [182]. ERα expression is significantly associated with PC poor survival [183]. ERα, acting as an oncogene, increased cell growth in two mouse models of aggressive PC, the PTEN-deficient and Hi-MYC mice, by stimulating PI3K and MAPK signaling pathway, MYC expression and altering glucose sensitivity [183]. ERβ expression was found at low levels or lost in PC and decreases in advanced PC [25,184]. Moreover, the ERβ2 and ERβ5—singularly and in co-expression—have prognostic value for PC progression; in fact, while ERβ1 expression decreases, the ERβ2 and ERβ5 expression increases in advanced PC [123]. Several data confirmed that ERβ play an anti-oncogenic role and it can be considered as a target to prevent PC development [121,185,186]. Interestingly, several in vitro studies showed that ERβ may be involved in programmed cell death regulation in PC (Table 2) (Figure 4).

In ER negative and androgen-independent DU-145 PC cells, the restore of ERβ expression strongly inhibited cell invasiveness and growth and triggered apoptotic mechanism as evidenced by Bax and cleaved caspase 3 and Parp-1 expression increase [29]. Proapoptotic actions of ERβ was demonstrated in androgen independent PC and also in BPH [187]. ERβ agonist induced extrinsic apoptotic mechanisms in prostatic stromal, luminal, and castrate-resistant basal epithelial cells of estrogen-deficient aromatase knock-out mice [187]. Furthermore, the observation that TNFα knock-out mice did not respond to ERβ agonist-mediated apoptotic effects confirmed that, in these models, apoptosis was androgen independent and TNFα mediated [187]. Similarly, ERβ agonist caused apoptosis in both in vivo PC xenografts models and in vitro AR-negative androgen independent cells lines PC3 and DU145, via the same extrinsic/caspase 8-mediated pathway activation [187]. Studies to understand the molecular events associated with CRPC identified an important role for the anti-apoptotic factor FLICE (or cFlip) [12] which is aberrantly expressed in high-grade PC and CRPC [194]. In vitro study performed in PC3 and DU145 and LNCaP prostate cancer cells provided evidence for ERβ-mediated transcriptional regulation of c-FLIP as an underlying mechanism in the development of CRPC [188]. While androgens inhibited apoptosis partly through transcriptional c-FLIP upregulation in androgen-dependent cells, they reduced c-FLIP expression in androgen-independent cells. Specifically, in PC3 and DU145 cells, the activation of androgen metabolism enzyme AKR1C1, catalyzed DHT transformation into 3β-Adiol which in turn, by activating ERβ, downregulated c-FLIP and induced apoptosis [188]. In another study, ERβ overexpression in PC3 and DU145 significantly inhibited cell proliferation and induced apoptosis [189]. Specifically, ERβ upregulation decreased TGFβ1 and IGF1 expression, while ERβ-selective antagonist PHTPP reversed this effects; the latter also increased Survivin and Bcl-2 expression levels [189]. Moreover, the use of TGFβ1 inhibitor LY2109761 downregulated the PHTPP-mediated effects on these protein expression [189]. All results confirmed how ERβ reduced androgen-independent prostate cancer cells’ viability and induced apoptosis through downregulation of anti-apoptotic proteins Survivin and Bcl-2 mediated by TGFβ1 and/or IGF1 signaling pathway [189]. In the same cell lines (PC3 and DU145), ERβ overexpression inhibited cells viability and migration and promoted apoptosis trough Bax and cleaved caspase 3 increase; furthermore, it reduced LPS-induced inflammation via downregulation of NF-κB signaling pathway [190]. ERβ-mediated apoptosis was also showed in both androgen-independent PC3 and 22Rv1 and androgen-dependent LNCaP cell lines [120]. Since these cells were isolated from PC that express low levels of ERβ (PC3 express very low levels of ERβ, whereas 22Rv1 cells are ERβ negative) to understand how ERβ causes apoptosis in prostate cancer cells, a doxycycline-regulated expression system has been developed for ERβ [120]. Results demonstrated that in both tested cells and also in AR + androgen dependent LNCaP that express low levels of ERβ, the use of ERβ-specific ligands 3β-Adiol, DPN, or 8β-VE2 activate apoptosis machinery [120]. This occurs by upregulation of FOXO3a and its downstream target PUMA via the intrinsic pathway as evidenced by Cyt c release, Bcl-2 decrease, and cleaved caspase 9 increase [120]. The same effect was demonstrated in PC3 xenograft model performed in nude mice [120].

### 3.3. Ovarian Cancer

OC represents a heterogeneous group of tumors characterized by specific risk factors, pathogenesis, molecular profiles, clinical course, and response to chemotherapy [195,196]. Major risk factors associated with OC include older age (postmenopausal women have a higher incidence) as well as positive family history of breast or ovarian cancers [197,198]. Most benign and malignant OC originates from one of three cell types: epithelial cells, stromal cells, and germ cells; more than 90% of OC malignant is of epithelial origin, 5–6% (e.g., granulosa cell tumors) originates from sex cord stromal cells while 2–3% (e.g., teratomas) from germ cells [199]. Malignant OC or carcinoma includes five main histological types: high-grade serous (70%), low-grade serous (<5%), endometrioid (10%), clear cell (10%), and mucinous (3%) [199]. Several mutations increase the risk of developing this cancer type. While TP53 and BRCA mutations are typical of high-grade serous carcinoma, those involving BRAF and KRAS genes frequently occur in low grade serous carcinoma. Endometrioid and clear cell carcinomas, frequently associated with endometriosis, are characterized by alterations of CTNNB1, PTEN, and POLE mutations, while clear cell carcinomas are characterized by ARID1A mutations. Mucinous carcinomas are rare forms associated with loss of CDKN2A copy number and KRAS mutations [196]. OC is currently one of the deadliest gynecological malignancies and this is attributed both to the diagnosis advanced tumor stage and to the rapid platinum-based chemotherapy resistance [195]. Therefore, new targets for early diagnosis, as well as better therapeutic options, are needed. Several epidemiological data suggest that etiology, pathogenesis, and progression of OC are related to estrogens exposure [200]. It has been reported that in OC, tumor-promoting estrogenic effects are mediated by both receptor-dependent and receptor-independent mechanisms [200]. Specifically, through ERα binding, they determine the transcriptional activation of specific target genes related to cell proliferation [200]. They can promote tumor progression by activating GPER-mediated ERK1/2 and PI3K signaling pathways [200]. Moreover, DNA mutagenic adducts can be formed from estrogens metabolism; accumulation of DNA mutations will lead to the neoplastic transformation of proliferating cells [200].

**Figure 5:**
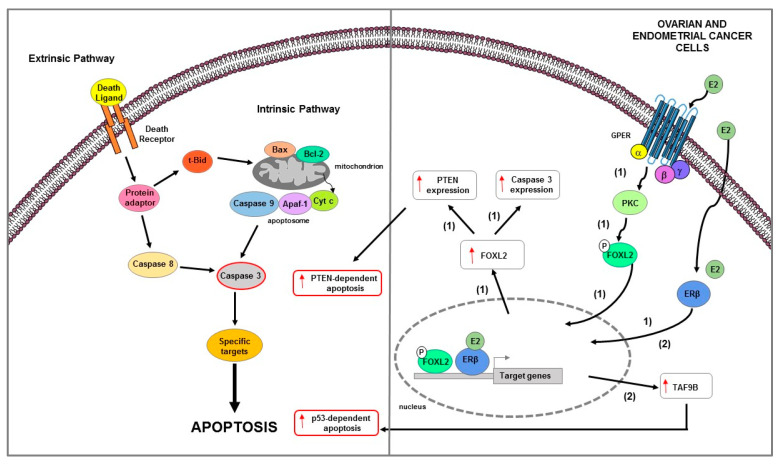
Main mechanisms of estrogen-induced apoptosis in ovarian and endometrial cancer cells. On the right side of the figure, the main transcriptional mechanisms mediated by ERs are indicated; on the left side, an illustration of apoptotic pathway (highlighted by the red outline) and its consequent activated is shown. ERβ genomic and GPER non genomic signaling are involved in apoptosis of OC cells (1). In EC cells (2) ERβ expression is associated with TAF9B mRNA increase. The red arrows pointing up indicate activation and/or expression increase. See text for more details. P: phosphate group.

However, ERβ was found to counteract the growth stimulating effects of ERα in OC cells [201]. ERβ expression is elevated in normal ovarian tissue, while it decreases during carcinogenesis [202]. Importantly, according to the comparison of normal ovarian tissue with OCs, it emerges both a decrease in ERβ mRNA expression and an increase in the ERα/ERβ mRNA ratio [202]. In agreement, complete loss of ERβ was observed in metastases of OC, while primary tumors showed low levels [203]. Furthermore, a meta-analysis study showed a positive correlation of ERβ expression with survival of OC patients; in particular, the overall survival of patients with tumors expressing cytoplasmic ERβ was significantly longer compared to those with ERβ-negative OC [204]. Expression of ERβ1, ERβ2, and ERβ4 (but not of ERβ5) was found to be decreased in OC, and this decrease correlated with ERβ gene promoter hypermethylation [205]. Hypermethylation is an event that causes a suppression of tumor suppressor genes and concomitant increase in the expression of oncogenes which drive tumorigenesis [206]. Moreover, in vitro studies support the tumor suppressive role of ERβ in the OC [191,201]. The effect of four ERβ agonists on proliferative behavior and gene expression in two OC cell lines, OVCAR-3 and OAW-42 cells, has been investigated [207]. Significant inhibitory effects on cell proliferation has been observed using all ERβ agonists; additionally, the ERβ knockdown increased OAW-42 cell proliferation [207]. In another study, the ERβ1 overexpression decreased SK-OV-3 ovarian growth and motility and activated apoptosis as shown by both caspase 3/7 activation and cell membrane phosphatidylserine translocation [191] (Table 2) (Figure 5).

The ERβ1-mediated cell motility and proliferation reduction was accompanied by extracellular matrix protein fibulin-1c increase and cyclin A2 decrease and p21 upregulation, respectively [191]. Recently, Wu J. and colleagues, using KGN human ovarian granulosa-like tumor cell line, clarified the mechanism by which estrogen feedback regulates FOXL2 to promote apoptosis [192]. It has been reported that FOXL2, a suppressor oncogenic factor, in the ovary induces cell death [208] and regulates the expression of factors involved in several signaling pathways such as TGFβ/BMP, MAPK, steroid synthesis, and PI3K/AKT [209]. The authors demonstrated that high doses of estrogen upregulated FOXL2 at both mRNA and protein levels via ERβ genomic pathway, reduced cell proliferation and induced apoptosis as evidenced by caspase 3 and PTEN mRNA expression increase [192]. Moreover, E2 treatment induced phosphorylation of FOXL2 via GPER/PKC non genomic signaling pathway [192]. Notably, FOXL2 deletion suppressed the proliferation inhibition and apoptosis induction mediated by estrogens; this suggested that E2 through FOXL2 regulated the expression of genes involved in both cell proliferation decrease and apoptosis increase [192]. Overall, these data suggested that FOXL2 might be beneficial in ovarian granulosa-like tumor cell line caused by abnormal estrogen [192].

### 3.4. Endometrial Cancer

EC is the most common gynecological cancer in western countries, affecting hundreds of thousands of women globally [210]. EC that originates from the lining of the uterus can be divided into two types: endometrioid (type 1), which affects approximately 80% of patients; and non-endometrioid (type 2), which affects approximately 20% of patients [211,212]. On the basis of the system of International Federation of Gynecology and Obstetrics (FIGO) which uses a scale grade from 1 to 3 based on relative glandular and solid tumor components [213], endometroid type ECs are divided into: grade 1 tumors with one component solid tumor less than 6%; grade 2, between 6% and 50%; and grade 3, more than 50% [211]. The first two subtypes are associated with a good prognosis, while the grade 3 subtype is associated with an intermediate to poor prognosis. Non-endometrioid EC, on the other hand, include mixed EC, UCS, CCEC, and SEC [211]. The last is the most common type and typically has a poor prognosis; however, the prognosis is worse for CCEC than SEC, whereas carcinosarcoma represents the high-grade form of metaplastic carcinoma. It has been reported that type 1 EC is estrogen dependent while type II is estrogen independent [214,215]. The first that arises from atypical hyperplasia and occurs most commonly before and around the time of menopause, is caused by excess estrogen following estrogen related pathway activation [214,216]. In EC, ER genomic binding is controlled by different and yet-unknown specific transcription factors and cofactors on which cell type-specific gene regulation depends. Relative excess estrogen, which represents the major risk factors for this form [212,217], can be caused by obesity, estrogen only HRT, and BC treatment with TAM, which acts as a partial ER agonist in endometrial cells. Type 2 EC that usually occurs in older and post-menopausal women is characterized by hormone-independent pathogenesis and unknown precursor lesions [217]. In the type 1 EC, the presence of ER and PR is positively associated with the prognosis of EC, including the survival rate and survival time [218]. The effects of progesterone are mediated through interaction with PR that leads to EC cell proliferation and invasion inhibition and apoptosis induction [219]. Indeed, endocrine therapy with progestin is the most commonly used together with their combination with TAM or aromatases inhibitors, showing similar response rates to progestogens alone [220]. The role of estrogen and ERs in estrogen-related EC has been extensively studied in recent years, but there are still a number of unresolved questions. In EC cells, there are close interactions between ERα and ERβ and the balanced co-expression of both receptors is a determining factor in EC carcinogenesis. ERs are expressed in the normal endometrium, although ERβ levels are lower than those of ERα [221,222]. In EC, ERβ is co-expressed with ERα and estrogenic effects occur predominantly through ERα [223]. Most studies indicated that there is no decrease in ERβ expression in EC and that it has tumor-promoting properties. Both ERβ1 and ERβ2 expression are unchanged in the EC compared to the postmenopausal endometrium [224,225] contrary to the results observed in ovarian [205] and breast cancers [226]. The presence of various ERβ splice variants was found in 46 endometrial tumors specimens and 28 normal endometrial tissues. In particular, four ERβ transcript variants was significantly elevated in cancer tissue and ERβ1, ERβ2, ERβ5, and five further variants expression was associated with the oncogenes MYBL2 or HER2 in EC [224].

According to few studies, ERβ can be considered a tumor suppressor in EC [193]. A study performed in 25 EC samples revealed a reduction in ERβ mRNA levels in tumor tissue compared to normal endometrium [227]. The expression analysis of ERβ1 and ERβ2 isoforms in 26 EC samples confirmed a decrease in ERβ2 levels in tumors compared to proliferative endometrium [228]. However, although there are no studies to date on estrogen-induced apoptosis via ERβ activation in EC, recent studies support its putative role as a tumor suppressor in endometrium [193] (Table 2) (Figure 5).

Results demonstrated that the downregulation of ERβ correlated with cell proliferation increase in ERα-/ERβ+ HEC-1A and ERα+/β+ cell line RL95/2 EC cell lines [193]. Furthermore, transcriptome analysis after ERβ silencing showed that this event was associated with both an increase in the expression of genes known to be upregulated in cancer and important for cell proliferation and a significant decrease in those related to cancer growth inhibition and apoptosis [193]. Specifically, among the apoptotic genes, the one that is heavily downregulated in HEC-1A cells after siRNA ERβ transfection is the TAF9B gene. TAF9B is a subunit of TFIID, which assists RNA polymerase II to initiate transcription [229]. This protein could participate in the regulation of cell apoptosis being a transcription coactivator for tumor suppressor p53 [230].

### 3.5. Role of ERs and GPER in the Apoptosis Regulation of Other Hormone-Related Cancers

#### 3.5.1. Testicular Cancer

TCs account for 1–1.5% of all male cancers [231]. They are distinguished in two large groups, the germ neoplasms (TGCTs) representing 95% of all testicular cancers and including seminoma and non-seminoma, and the rarer non germ neoplasms including LCT, Sertoli cell tumor and gonadoblastoma [231]. Although the androgen dependence of testicular function—including germ cell development—is well known, the observation that fetal xenoestrogen exposure may contribute to testicular germ cell carcinogenesis [232] suggests an estrogen-dependence for TGCTs. It has been reported that estrogens play a central role in the regulation of both normal testicular functions and in testicular cancer [233,234]. In the testis, physiological effects of estrogens are mediated not only by classical ERα and ERβ, but also GPER by activating both genomic and non-genomic pathways that can work independently or cooperate to regulate the same molecular event [234,235]. Testicular ERα and ERβ expression is highly variable, with major differences between species [236]. GPER is also expressed in germ cells (spermatogonia, spermatocytes, spermatids, spermatozoa) and somatic cells (telocytes, peritubular, Sertoli, and Leydig cells) [233]. Estrogen actions on spermatogenesis influence, in a cell-specific manner, germ cell proliferation, differentiation, as well as germ cell survival and apoptosis. Notably, apoptosis is a molecular event that is part of the regulation of normal spermatogenesis useful for maintaining the correct number of spermatogenic cells which are supported by the Sertoli cells. In recent decades, the role of GPER on testicular functions [233] including physiological responses [237,238,239,240] and testicular tumors [241] has been explored. In particular, it has been reported that in testicular tubular compartment, GPER mediates estrogen action on both somatic and germ cells. The reduced GPER expression in peritubular cell seems to be associated to infertility [242], while it is involved in the maintenance of Sertoli cell number and consequently for normal testis development and homeostasis [243,244,245,246]. In germ cells through a ERs crosstalk [237,238,239,247], it modulates the proliferation of spermatogonia [247,248,249] and the physiological apoptosis regulating spermatocytes [238,239,250] and spermatids number [237]. Furthermore, in testicular interstitial compartment, GPER play important roles in regulating estrogen-dependent lipid homeostasis in Leydig cells [251] and testosterone biosynthesis [252], as well as the number and physiology of telocytes [253,254] that contribute to maintain lipid balance.

**Figure 6:**
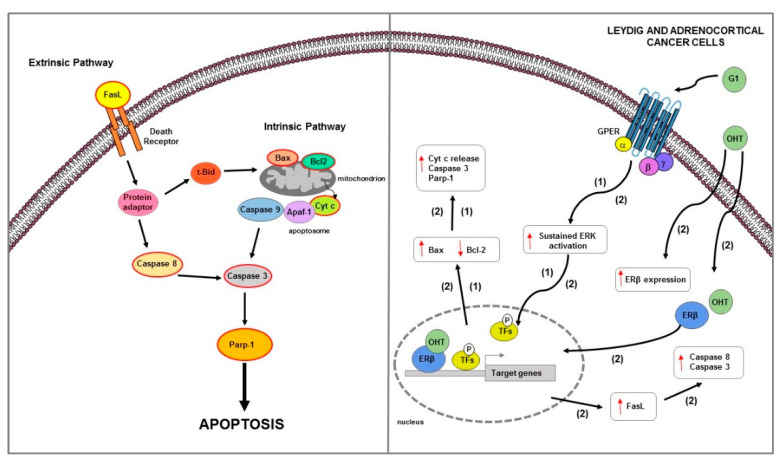
Main mechanisms of estrogen-induced apoptosis in Leydig and adrenocortical cancer cells. On the right side of the figure, the main transcriptional mechanisms mediated by ERs are indicated; on the left side, an illustration of how the apoptotic pathway (highlighted by the red outline) consequently activated is shown. GPER activation by G1 triggers a mitochondrion-dependent apoptotic pathway in both LCT (1) and ACC (2) cells. In ACC (2), OHT, by ERβ upregulation, also increases the FasL expression and caspase 8 and 3 activation. The red arrows pointing up indicate activation and/or expression increase, while those pointing down indicate inhibition and/or expression decrease. See text for more details. P: phosphate group.

The role of ERs and GPER in testicular cancer has been also reported [233]. In particular, a tumor promotion or suppression role was confirmed for ERα and ERβ, respectively, while for GPER—depending on the testicular tumor type—an involvement in both progression and cell death has been demonstrated. In the seminoma TCAM2 cell line lacking ERα, the ERβ activation caused cell necroptosis and autophagy [255]. Meanwhile, in the JKT-1 seminoma cells expressing GPER and ERβ—but not ERα—the use of E2 [256], BPA [257,258], and G1 [259] increased cell proliferation through a rapid activation of ERK1/2, PKA [256,257], and PKG [257] signaling pathway. On the other hand, the E2-dependent activation of ERβ [260] or the GPER antagonist G15 [259] reduced JKT-1 cell growth. It has been observed that GPER overexpression was associated with ERβ downregulation in both human testicular carcinoma in situ and seminomas. In fact, in a study performed in TCam-2 cells, E2—through a GPER/PKA/CREB signaling pathway—determined an increase in cell proliferation by inducing ERα36 expression [261]. Furthermore, E2-dependent activation of the GPER/ERK/c-Fos pathway reduced ERβ expression in the same cells [262]. Conversely, in LCT where ERα is overexpressed, GPER activation caused a marked reduction in cell growth in vitro and in vivo [38]. In particular, in the R2C LCT, GPER activation by G1 triggered a mitochondrion-dependent apoptotic pathway [38]. This event required a prolonged activation of ERK1/2 followed by DNA fragmentation, Bcl-2 decrease, Bax increase, Cyt c release, and caspase 3 and Parp-1 activation [38] (Figure 6). Therefore, the fact that high GPER levels correlated with the low ones of ERβ [262] suggested a potential therapeutic role of GPER inhibitors for testicular carcinoma in situ and seminomas treatment. Furthermore, GPER activation by selective ligands led to opposite results in seminoma and in LCT; this observation demonstrates a cell specificity of estrogen-dependent testicular tumorigenesis.

#### 3.5.2. Adrenocortical Cancer

ACC is a rare and highly malignant tumor associated with a poor prognosis [263]. Complex pathogenesis and limited therapeutic options are characteristic of this aggressive neoplasm. Genomic characterizations of ACC that identified a correlation between tumor onset and several genetic mutations—including TP53, CTNNB1, IGF2, PRKAR1A, RPL22, TERF2, CCNE1, and NF1 genes [264,265]—revealed high heterogeneity and histotype-specific genomic profiles [266]. Early diagnosis followed by tumor surgical excision, associated with mitotane administered alone or in combination with chemotherapy drugs, represents the only possibility of cure for ACC patients [267]. Despite its wide use, mitotane presents many limitations—mainly due to its toxicity, narrow therapeutic window, and its numerous side effects [267].

Epidemiological and experimental studies suggest a possible involvement of estrogens in the development of ACC. Adrenal tumors are reported to be found more frequently in women than in men [268]. Furthermore, the use of estrogen-progestins represents a risk factor for the adrenal carcinomas development [269]. It has been largely demonstrated that estrogens effects on adrenal gland are mediated by ERs that are differently expressed in normal and neoplastic adrenal cortex [270]. In the human fetal adrenal gland, the mRNA of ERβ is much more expressed than that of ERα [271,272]; ERβ is detected mainly in human adult adrenal tissues [270] and in the definite zone of the adrenal cortex at prepubertal age [273]. In ACC, ERs expression is questionable and controversial. Some data from immunohistochemical studies reported a negativity for ERα and an increase for ERβ expression [270]; by contrast, other studies reported a higher ERα expression respect to ERβ in ACC [274]. Moreover, Barzon et al. showed an increased aromatase activity in ACC, hypothesizing a paracrine estrogenic effect in this tumor [274]. In ACC, ERα acts as an oncogene; its activation may occur by an E2-dependent mechanism or alternatively by IGF2/IGF1R in a ligand-independent manner, by activating the IGF1R/AKT proliferative pathways [275]. Furthermore, the use of hydroxytamoxifen, an active metabolite of the estrogen antagonist TAM, reduced IGF1R expression levels and E2 and IGF2-mediated cell proliferation increase in both in vitro and in an ACC xenograft model [275]. Another in vitro study demonstrated that physiological concentrations of E2 stimulated H295R cell growth, while the treatment with OHT, by increasing the pro-apoptotic factor FasL expression and caspase 8 and 3 activation, reduced H295R cell proliferation through ERβ upregulation [276] (Figure 6). It is well known that TAM and its active metabolite OHT in addition to antiestrogenic activity [276] also work as agonist of GPER [277]. In ACC, GPER activation determined a growth inhibitory effect on both in vitro and in vivo xenograft models [40]. Specifically—in H295R cells—G1 caused cell cycle arrest, DNA damage, and apoptotic cell death as evidenced by DNA fragmentation; Bcl-2 decrease; Bax, cytosolic Cyt c, and cleaved Parp-1 increase [40]; these events required a sustained ERK activation which is known to be involved in apoptosis [239,278] (Figure 6).

Estrogen-mediated apoptosis was demonstrated in SW13 adrenocortical cell line [279]. In this cell model, high doses of E2 and progesterone have inhibitory on cell proliferation as evidenced by CB1 and CD1 expression decrease and G2/M cell cycle arrest [279]. Moreover, sub-G1 apoptosis was confirmed by fragmented and condensed nuclear chromatin staining [279]. The same authors, in another work, demonstrated that in SW13 cells, E2 and only ERα specific agonist PPT, but not specific agonist for ERβ, were able to induce apoptosis [280].

The conflicting results obtained in the above studies are probably due to different doses of E2 used as well as to use of two different experimental models of ACC that have a specific histological differentiation degree and endocrine characteristics [281]. H295R cells derive from a female affected by a primary adrenocortical carcinoma and possess the biochemical capacity to synthesize different classes of steroid hormones, including glucocorticoids, mineralocorticoids and androgens and are responsive to pituitary ACTH and AngII. On the other hand, SW-13 cells are a depot in the adrenal of a primary lung cancer and have a reduced secretion capacity of steroid hormones and derived from a stage IV adrenocortical carcinoma [281].

Altogether, since literature data concerning ERs expression and functions in ACC are still limited, further studies are necessary to better clarify and define ERs role in mediating apoptotic events in this tumor.

## 4. Conclusions

Estrogens are important regulators of cell proliferation in many reproductive and extra-reproductive tissues in both sexes. Generally, they stimulate growth and inhibit apoptosis through ER-mediated mechanisms in many cancer cell types. However, it has been reported that, in some BCs, high-doses of estrogens can be effective for the treatment of advanced or resistant to antiestrogens tumors (estrogen paradox). It has been suggested that BC cells, after a long period of estrogen deprivation, can adapt to low levels of estrogens by enhancing their sensitivity to E2 on which depends the activation of apoptotic mechanisms. The estrogen-mediated cell proliferation control is cell-specific and depends on the expression of both ERα and ERβ and its relative several splice variants as well as GPER. There is clear evidence that ERα mediates the proliferative effects of estrogens in several hormone-dependent tumors—such as breast, prostate, ovarian, endometrial, testicular, and adrenocortical cancers. E2 binding ERα can activate genomic and non-genomic signaling involved in both cell cycle progression and apoptotic cascade prevention.

By contrast, E2 binding ERβ directs cells to death by apoptosis. Interestingly, ERβ, when co-expressed with ERα, acts as a brake on ERα-mediated proliferative effects and activates both extrinsic and intrinsic apoptotic mechanisms in several hormone-dependent cancers. According to these notions, a progressive decline of ERβ expression has been reported during the development of breast, prostate, and ovarian tumors. Although unbalanced ERβ expression could play a pivotal role in the progression of many cancer types, its prognostic role remains controversial in some cancers. Indeed, in order to better define the role of ERβ in cancer, it would be necessary to evaluate the expression levels of its various isoforms which could, therefore, clarify some contradictory results that correlate ERβ expression with a better or poor clinical outcome. Moreover, at least in certain types of cancers, the complete profile of both ERs as well as GPER needs to be evaluated. It has been observed that GPER overexpression (e.g., testicular carcinoma in situ and seminomas) is associated with ERβ downregulation and mediates proliferative effects. By contrast, in some tumors (e.g., LCT or ACC) where ERα is overexpressed, GPER activation causes a marked reduction in cell growth and apoptosis.

In conclusion, the potential usefulness of ERs or GPER as therapeutic targets in some cancers should be evaluated in prospective clinical trials. In this regard, the development of specific ligands capable of triggering apoptotic mechanisms may open new perspectives for the study of alternative treatments in hormone-dependent tumors.

## Figures and Tables

**Table 1:** Molecular mechanisms activated by ERs and involved in BC cells apoptosis regulation.

Cancer Cell Types	Molecular Mechanisms	References
MCF-7RLX	Fas increase HER2/neu inhibition NF-κB inhibition	[153]
MCF-7TAMLT	Fas increase HER2/neu inhibition NF-κB inhibition	[154]
MCF-7FR and LTED	Bim increase JNK signaling activation Parp-1 cleavage P53 signaling activation UPR activation	[156]
LTED	Caspase activation DNA fragmentation Fas/FasL signaling pathway activation	[146]
LTED and E8CASS	Bcl-2 decrease Caspase 9 and 7 activation NF-KB decrease	[157]
E8CASS	DNA degradation E9 mRNA increase	[158]
MCF-7:5C	Bak, Bax, Bim increase Caspase 7 and 9 activation Cyt c release Parp-1 cleavage P53 increase	[159]
MCF-7:5C	Bax and Bim increase Caspase 4 increase ERS activation IFN, IL, and arachidonic acid-related genes increase Parp-1 cleavage UPR activation	[160]
MCF-7:5C	DNA fragmentation IFITM1 decrease Parp-1 cleavage JAK/STAT-mediated P21 increase	[161]
MCF-7:5C	DNA fragmentation IFITM1 decrease MUC1 decrease	[126]
MCF-7:5C	ERα/cSrc interaction activation ERS and mitochondrial stress activation Extrinsic and intrinsic apoptotic pathways activation HO-1 increase ROS increase UPR activation	[162]
MCF-7:5C	Apoptosis induction PERK/ STAT3/NF-κB /TNFα signaling pathway activation	[163]
MCF-7:5C	ATF4 increase CHOP increase CReP inhibition eIF2α phosphorylation increase GADD34 decrease Parp-1 cleavage PERK increase	[127]
MCF-7	ERα/p53 interaction decrease ERβ/p53 interaction increase Increase of ERβ/p53-mediated DR5 and Bax transcription	[134]

**Table 2:** Molecular mechanisms activated by ERs and involved in other hormone-dependent cancer cells apoptosis regulation.

Cancer Cell Types	Molecular Mechanisms	References
Prostate		
DU-145	Bax increase Cleaved caspase 3 increase Parp-1 increase	[29]
PC3 and DU-145	Caspase 8 activation Extrinsic apoptotic pathway increase TNFα increase	[187]
PC3 and DU-145	AKR1C1 activation c-FLIP decrease	[188]
PC3 and DU-145	Bcl-2 decrease Survivin decrease TGFβ1/IGF1 signaling inhibition	[189]
PC3 and DU-145	Bax increase Cleaved caspase 3 increase	[190]
PC3, 22Rv1 and LNCaP	Bcl-2 decrease Cleaved caspase 9 increase Cyt c release FOXO3A increase PUMA increase	[120]
Ovarian		
SK-OV-3	Caspase 3 and 7 activation Membrane phosphatidylserine traslocation	[191]
KNG	Caspase 3 increase FOXL2 increase PTEN increase	[192]
Endometrial		
HEC-1A	TAF9B increase	[193]

## Abbreviations

3β-Adiol	5α-androstane-3β,17β-diol
8β-VE2	8-vinylestra-1:3,5 (10)-triene-3,17β-diol
AC	adenylate cyclase
ACC	adrenocortical cancer
ACTH	adrenocorticotropic hormone
ADT	androgen deprivation therapy
AF-1	transcriptional activation domain 1
AF-2	transcriptional activation domain 2
AI	aromatase inhibitors
AKR1C1	aldo-keto reductase family 1 member C1
AKT	protein kinase B
ANGII	angiotensin II
AR +	androgen receptor positive
AR	androgen receptor
ARID1A	AT-rich interactive domain-containing protein 1A
ATF4	activating transcription factor 4
ATF6	activating transcription factor 6
ATM	ataxia telangiectasia
AVPC	aggressive variant prostate cancer
BC	breast cancer
BCL-2	B-cell lymphoma-2
BCSCs	breast cancer stem cells
BH	Bcl-2 homology
BH3	BCL-2 homology 3
BMP	bone morphogenic protein
BPA	bisphenol A
BPH	benign prostatic hyperplasia
BRAF	serine/threonine-protein kinase B-raf
BRCA1	BReast CAncer gene 1
BRCA2	BReast CAncer gene 2
C/EBPs	CCAAT/enhancer binding proteins
Ca^2+^	calcium ion
cAMP	cyclic adenosine monophosphate
CB1	cyclin B1
CCEC	clear cell carcinoma
CCNE1	cyclin E1
CD1	cyclin D1
CDH1	E-cadherin gene
CDK4	cyclin dependent kinase 4
CDKN2A	cyclin dependent kinase inhibitor 2a
c-FLIP	FLICE/caspase-8-inhibitory protein
CHOP	C/EBP homologous protein
CREB	cAMP response element-binding protein
CReP	constitutive repressor of eIF2a phosphorylation
CRPC	castration resistant PC
CTNNB1	catenin beta 1
CYT C	cytochrome c
DHT	dihydrotestosterone
DPN	2:3-bis (4-hydroxyphenyl) propionitrile
DR4 or 5	death receptor 4 or 5
E2	estradiol
EAB	estrogen and androgen blocking
EC	endometrial cancer
ECM	extracellular matrix
EGFR	epidermal growth factor receptor
eIF2a	initiation factor 2 alpha
EMT	epithelial–mesenchymal transition
ER -	ER negative
ER	estrogen receptor
ER+	ER positive
ERE	estrogen response element
ERK	extracellular signal-regulated kinase
ERK1/2	extracellular signal-regulated kinase 1
ERs	estrogen receptors
ERS	reticulum endoplasmic stress
ERα	estrogen receptor alpha
ERβ	estrogen receptor beta
ESR1	estrogen receptor 1
ESR2	estrogen receptor 2
FADD	Fas-associated protein with death domain
FAS	fas cell surface death receptor
FASL	FAS ligand
FLICE	FADD (Fas-associated death domain)-like IL-1β–converting enzyme
C-FLIP	cellular FLICE inhibitory protein
FOXL2	forkhead box protein L2
FOXO3a	forkhead box O3a
FSH	follicle-stimulating hormone
GADD34	growth-arrest- and DNA-damage-induced transcript 34
GPCR	G-protein-coupled receptor
GPER	G protein-coupled estrogen receptor 1
H3K4me3	histone H3 trimethylation of lysine 4
H3K9me3	histone H3 trimethylation of lysine 9
HER2	human epidermal growth factor receptor 2
Hi-Myc	human c-Myc driven prostate cancer
HO-1	heme oxygenase 1
HRT	hormone replacement therapy
IAP	inhibitor apoptosis protein
IFITM1	IFN-induced transmembrane protein 1
IFNα	interferon α
IGF1	insulin like growth factor 2
IGF1R	insulin-like growth factor 1 receptor
IGF2	insulin like growth factor 2
IRE1-α	inositol-requiring protein 1 alpha
JAK	janus kinase
JNK	c-Jun N-terminal kinase
KRAS	kirsten rat sarcoma virus
LCT	Leydig cell tumor
LH	luteinizing hormone
LHRH	luteinizing hormone releasing hormone
LTED	long-term estrogen-deprived
LPS	lipopolysaccharide
MAPK	mitogen-activated protein kinase
MEK	MAP kinase kinase
MYBL2	v-myb myeloblastosis viral oncogene homolog (avian)-like 2
MPs	matrix metalloproteinases
mTOR	mammalian target of rapamycin
MUC1	mucin 1
N-CoR	nuclear receptor co-repressor 1
NF1	neurofibromatosis type 1
NF-κB	nuclear factor kappa B
NO	nitric oxide
OC	ovarian cancer
OHT	hydroxytamoxifen
PARP-1	poly (ADP-ribose) polymerase 1
PC	prostate cancer
PDGFA	platelet derived growth factor subunit A
PERK	protein kinase RNA (PKR)-like endoplasmic reticular (ER)
PHTPP	4-[2-Phenyl-5:7-bis(trifluoromethyl)pyrazolo [1,5-a] pyrimidin-3-yl] phenol
PI3K	phoshatidylinositol-3 kinase
PKA	protein kinase A
PKC	protein kinase C
PKG	protein kinase G
PLC	phospholipase C
POLE	DNA polymerase epsilon, catalytic subunit
PPT	1:3,5-tris (4-hydroxyphenyl)-4-propyl-1H-pyrazole
PR	progesterone receptor
PR-A	progesterone receptor isoform A
PR-B	progesterone receptor isoform B
PRKAR1A	protein kinase cAMP-dependent regulatory type I alpha
PTEN	phosphatase and tensin homolog
TFs	transcription factors
PUMA	p53 upregulated modulator of apoptosis
PXN	paxillin
Rb	retinoblastoma protein
RLX	raloxifene
ROS	reactive oxygen species
RPL22	ribosomal protein L22
SEC	serous carcinoma
SERMs	selective ER modulators
SFKs	Src family kinases
SHBG	sex hormone binding globulin
SMRT	silencing mediator of retinoic acid and thyroid hormone receptor
STAT	signal transducer and activator of transcription
STK11	serine/threonine kinase 11
SUV39H1	suppressor of variegation 3-9 homolog 1
SUV39H2	suppressor of variegation 3-9 homolog 2
TAF9B	TATA-Box Binding Protein Associated Factor 9
TAM	tamoxifen
TCs	testicular cancers
TERF2	telomere specific protein 2
TFIID	transcription factor IID
TGCTs	testicular germ cell tumors
TGFβ1	transforming growth factor beta-1
TNBC	triple negative breast cancers
TNF	tumor necrosis factor
TNF-R1	tumor necrosis factor-receptor 1
TRADD	tumor necrosis factor receptor type 1-associated death domain protein
TRAIL	TNF-related apoptosis-inducing ligand
TRAIL	TNF-related apoptosis-inducing ligand
TRAILR1 or -R2	TRAIL receptor
TUNEL	Terminal deoxynucleotidyl transferase dUTP nick end labeling
UCS	endometrial uterine carcinosarcoma
UPR	unfolded protein response
VEGFA	vascular endothelial growth factor A

## References

[1] Ke F.F.S., Vanyai H.K., Cowan A.D., Delbridge A.R.D., Whitehead L., Grabow S., Czabotar P.E., Voss A.K., Strasser A. (2018). Embryogenesis and Adult Life in the Absence of Intrinsic Apoptosis Effectors BAX, BAK, and BOK. Cell.

[2] Codispoti B., Makeeva I., Sied J., Benincasa C., Scacco S., Tatullo M. (2019). Should we reconsider the apoptosis as a strategic player in tissue regeneration?. Int. J. Biol. Sci..

[3] Arandjelovic S., Ravichandran K.S. (2015). Phagocytosis of apoptotic cells in homeostasis. Nat. Immunol..

[4] Carneiro B.A., El-Deiry W.S. (2020). Targeting apoptosis in cancer therapy. Nat. Rev. Clin. Oncol..

[5] Lindsten T., Ross A.J., King A., Zong W.X., Rathmell J.C., Shiels H.A., Ulrich E., Waymire K.G., Mahar P., Frauwirth K. (2000). The combined functions of proapoptotic Bcl-2 family members bak and bax are essential for normal development of multiple tissues. Mol. Cell.

[6] Hacker G. (2000). The morphology of apoptosis. Cell Tissue Res..

[7] Hengartner M.O. (2000). The biochemistry of apoptosis. Nature.

[8] Elmore S. (2007). Apoptosis: A review of programmed cell death. Toxicol. Pathol..

[9] Jin Z., El-Deiry W.S. (2005). Overview of cell death signaling pathways. Cancer Biol. Ther..

[10] Boice A., Bouchier-Hayes L. (2020). Targeting apoptotic caspases in cancer. Biochim. Biophys. Acta Mol. Cell Res..

[11] Nicholson D.W. (1999). Caspase structure, proteolytic substrates, and function during apoptotic cell death. Cell Death Differ..

[12] Safa A.R. (2012). c-FLIP, a master anti-apoptotic regulator. Exp. Oncol..

[13] Kale J., Osterlund E.J., Andrews D.W. (2018). BCL-2 family proteins: Changing partners in the dance towards death. Cell Death Differ..

[14] Fridman J.S., Lowe S.W. (2003). Control of apoptosis by p53. Oncogene.

[15] Pfeffer C.M., Singh A.T.K. (2018). Apoptosis: A Target for Anticancer Therapy. Int. J. Mol. Sci..

[16] Fernald K., Kurokawa M. (2013). Evading apoptosis in cancer. Trends Cell Biol..

[17] Jan R., Chaudhry G.E. (2019). Understanding Apoptosis and Apoptotic Pathways Targeted Cancer Therapeutics. Adv. Pharm. Bull..

[18] Shirley S., Micheau O. (2013). Targeting c-FLIP in cancer. Cancer Lett..

[19] Fulda S., Debatin K.M. (2002). IFNgamma sensitizes for apoptosis by upregulating caspase-8 expression through the Stat1 pathway. Oncogene.

[20] Amarante-Mendes G.P., Griffith T.S. (2015). Therapeutic applications of TRAIL receptor agonists in cancer and beyond. Pharmacol. Ther..

[21] Ngoi N.Y.L., Choong C., Lee J., Bellot G., Wong A.L.A., Goh B.C., Pervaiz S. (2020). Targeting Mitochondrial Apoptosis to Overcome Treatment Resistance in Cancer. Cancers.

[22] Song R.X., Santen R.J. (2003). Apoptotic action of estrogen. Apoptosis.

[23] Paterni I., Granchi C., Katzenellenbogen J.A., Minutolo F. (2014). Estrogen receptors alpha (ERalpha) and beta (ERbeta): Subtype-selective ligands and clinical potential. Steroids.

[24] Yasar P., Ayaz G., User S.D., Gupur G., Muyan M. (2017). Molecular mechanism of estrogen-estrogen receptor signaling. Reprod. Med. Biol..

[25] Horvath L.G., Henshall S.M., Lee C.S., Head D.R., Quinn D.I., Makela S., Delprado W., Golovsky D., Brenner P.C., O’Neill G. (2001). Frequent loss of estrogen receptor-beta expression in prostate cancer. Cancer Res..

[26] Roger P., Sahla M.E., Makela S., Gustafsson J.A., Baldet P., Rochefort H. (2001). Decreased expression of estrogen receptor beta protein in proliferative preinvasive mammary tumors. Cancer Res..

[27] Bardin A., Boulle N., Lazennec G., Vignon F., Pujol P. (2004). Loss of ERbeta expression as a common step in estrogen-dependent tumor progression. Endocr. Relat. Cancer.

[28] Hall J.M., McDonnell D.P. (1999). The estrogen receptor beta-isoform (ERbeta) of the human estrogen receptor modulates ERalpha transcriptional activity and is a key regulator of the cellular response to estrogens and antiestrogens. Endocrinology.

[29] Cheng J., Lee E.J., Madison L.D., Lazennec G. (2004). Expression of estrogen receptor beta in prostate carcinoma cells inhibits invasion and proliferation and triggers apoptosis. FEBS Lett..

[30] Strom A., Hartman J., Foster J.S., Kietz S., Wimalasena J., Gustafsson J.A. (2004). Estrogen receptor beta inhibits 17beta-estradiol-stimulated proliferation of the breast cancer cell line T47D. Proc. Natl. Acad. Sci. USA.

[31] Acconcia F., Totta P., Ogawa S., Cardillo I., Inoue S., Leone S., Trentalance A., Muramatsu M., Marino M. (2005). Survival versus apoptotic 17beta-estradiol effect: Role of ER alpha and ER beta activated non-genomic signaling. J. Cell. Physiol..

[32] Welboren W.J., Sweep F.C., Span P.N., Stunnenberg H.G. (2009). Genomic actions of estrogen receptor alpha: What are the targets and how are they regulated?. Endocr. Relat. Cancer.

[33] Levin E.R. (2009). Membrane oestrogen receptor alpha signalling to cell functions. J. Physiol..

[34] Marino M., Ascenzi P. (2008). Membrane association of estrogen receptor alpha and beta influences 17beta-estradiol-mediated cancer cell proliferation. Steroids.

[35] Matthews J., Gustafsson J.A. (2003). Estrogen signaling: A subtle balance between ER alpha and ER beta. Mol. Interv..

[36] Thomas P., Pang Y., Filardo E.J., Dong J. (2005). Identity of an estrogen membrane receptor coupled to a G protein in human breast cancer cells. Endocrinology.

[37] Filardo E.J., Quinn J.A., Bland K.I., Frackelton A.R. (2000). Estrogen-induced activation of Erk-1 and Erk-2 requires the G protein-coupled receptor homolog, GPR30, and occurs via trans-activation of the epidermal growth factor receptor through release of HB-EGF. Mol. Endocrinol..

[38] Chimento A., Casaburi I., Bartucci M., Patrizii M., Dattilo R., Avena P., Ando S., Pezzi V., Sirianni R. (2013). Selective GPER activation decreases proliferation and activates apoptosis in tumor Leydig cells. Cell Death Dis..

[39] Jung J. (2019). Role of G Protein-Coupled Estrogen Receptor in Cancer Progression. Toxicol. Res..

[40] Chimento A., Sirianni R., Casaburi I., Zolea F., Rizza P., Avena P., Malivindi R., De Luca A., Campana C., Martire E. (2015). GPER agonist G-1 decreases adrenocortical carcinoma (ACC) cell growth in vitro and in vivo. Oncotarget.

[41] Ascenzi P., Bocedi A., Marino M. (2006). Structure-function relationship of estrogen receptor alpha and beta: Impact on human health. Mol. Asp. Med..

[42] Miller M.M., McMullen P.D., Andersen M.E., Clewell R.A. (2017). Multiple receptors shape the estrogen response pathway and are critical considerations for the future of in vitro-based risk assessment efforts. Crit. Rev. Toxicol..

[43] Gibson D.A., Saunders P.T. (2012). Estrogen dependent signaling in reproductive tissues—A role for estrogen receptors and estrogen related receptors. Mol. Cell. Endocrinol..

[44] Murphy A.J., Guyre P.M., Wira C.R., Pioli P.A. (2009). Estradiol regulates expression of estrogen receptor ERalpha46 in human macrophages. PLoS ONE.

[45] Li L., Haynes M.P., Bender J.R. (2003). Plasma membrane localization and function of the estrogen receptor alpha variant (ER46) in human endothelial cells. Proc. Natl. Acad. Sci. USA.

[46] Denger S., Reid G., Kos M., Flouriot G., Parsch D., Brand H., Korach K.S., Sonntag-Buck V., Gannon F. (2001). ERalpha gene expression in human primary osteoblasts: Evidence for the expression of two receptor proteins. Mol. Endocrinol..

[47] Wang Z., Zhang X., Shen P., Loggie B.W., Chang Y., Deuel T.F. (2005). Identification, cloning, and expression of human estrogen receptor-alpha36, a novel variant of human estrogen receptor-alpha66. Biochem. Biophys. Res. Commun..

[48] Leygue E., Murphy L.C. (2013). A bi-faceted role of estrogen receptor beta in breast cancer. Endocr. Relat. Cancer.

[49] Shaaban A.M., Green A.R., Karthik S., Alizadeh Y., Hughes T.A., Harkins L., Ellis I.O., Robertson J.F., Paish E.C., Saunders P.T. (2008). Nuclear and cytoplasmic expression of ERbeta1, ERbeta2, and ERbeta5 identifies distinct prognostic outcome for breast cancer patients. Clin. Cancer Res..

[50] Leung Y.K., Mak P., Hassan S., Ho S.M. (2006). Estrogen receptor (ER)-beta isoforms: A key to understanding ER-beta signaling. Proc. Natl. Acad. Sci. USA.

[51] Ogawa S., Inoue S., Watanabe T., Hiroi H., Orimo A., Hosoi T., Ouchi Y., Muramatsu M. (1998). The complete primary structure of human estrogen receptor beta (hER beta) and its heterodimerization with ER alpha in vivo and in vitro. Biochem. Biophys. Res. Commun..

[52] Omoto Y., Eguchi H., Yamamoto-Yamaguchi Y., Hayashi S. (2003). Estrogen receptor (ER) beta1 and ERbetacx/beta2 inhibit ERalpha function differently in breast cancer cell line MCF7. Oncogene.

[53] Mal R., Magner A., David J., Datta J., Vallabhaneni M., Kassem M., Manouchehri J., Willingham N., Stover D., Vandeusen J. (2020). Estrogen Receptor Beta (ERbeta): A Ligand Activated Tumor Suppressor. Front. Oncol..

[54] Puranik N.V., Srivastava P., Bhatt G., John Mary D.J.S., Limaye A.M., Sivaraman J. (2019). Determination and analysis of agonist and antagonist potential of naturally occurring flavonoids for estrogen receptor (ERalpha) by various parameters and molecular modelling approach. Sci. Rep..

[55] Bafna D., Ban F., Rennie P.S., Singh K., Cherkasov A. (2020). Computer-Aided Ligand Discovery for Estrogen Receptor Alpha. Int. J. Mol. Sci..

[56] Jin J., Wu P., Zhang X., Li D., Wong W.L., Lu Y.J., Sun N., Zhang K. (2020). Understanding the interaction of estrogenic ligands with estrogen receptors: A survey of the functional and binding kinetic studies. J. Environ. Sci. Health C Toxicol..

[57] Farzaneh S., Zarghi A. (2016). Estrogen Receptor Ligands: A Review (2013–2015). Sci. Pharm..

[58] Maekawa R., Sato S., Okada M., Lee L., Tamura I., Jozaki K., Kajimura T., Asada H., Yamagata Y., Tamura H. (2016). Tissue-Specific Expression of Estrogen Receptor 1 Is Regulated by DNA Methylation in a T-DMR. Mol. Endocrinol..

[59] Saunders P.T., Millar M.R., Williams K., Macpherson S., Harkiss D., Anderson R.A., Orr B., Groome N.P., Scobie G., Fraser H.M. (2000). Differential expression of estrogen receptor-alpha and -beta and androgen receptor in the ovaries of marmosets and humans. Biol. Reprod..

[60] Taylor A.H., Al-Azzawi F. (2000). Immunolocalisation of oestrogen receptor beta in human tissues. J. Mol. Endocrinol..

[61] Couse J.F., Lindzey J., Grandien K., Gustafsson J.A., Korach K.S. (1997). Tissue distribution and quantitative analysis of estrogen receptor-alpha (ERalpha) and estrogen receptor-beta (ERbeta) messenger ribonucleic acid in the wild-type and ERalpha-knockout mouse. Endocrinology.

[62] Kelly M.J., Levin E.R. (2001). Rapid actions of plasma membrane estrogen receptors. Trends Endocrinol. Metab..

[63] Nilsson S., Makela S., Treuter E., Tujague M., Thomsen J., Andersson G., Enmark E., Pettersson K., Warner M., Gustafsson J.A. (2001). Mechanisms of estrogen action. Physiol. Rev..

[64] Pedram A., Razandi M., Aitkenhead M., Hughes C.C., Levin E.R. (2002). Integration of the non-genomic and genomic actions of estrogen. Membrane-initiated signaling by steroid to transcription and cell biology. J. Biol. Chem..

[65] Bjornstrom L., Sjoberg M. (2005). Mechanisms of estrogen receptor signaling: Convergence of genomic and nongenomic actions on target genes. Mol. Endocrinol..

[66] Katzenellenbogen B.S., Katzenellenbogen J.A. (2000). Estrogen receptor transcription and transactivation: Estrogen receptor alpha and estrogen receptor beta: Regulation by selective estrogen receptor modulators and importance in breast cancer. Breast Cancer Res..

[67] Shim W.S., Conaway M., Masamura S., Yue W., Wang J.P., Kmar R., Santen R.J. (2000). Estradiol hypersensitivity and mitogen-activated protein kinase expression in long-term estrogen deprived human breast cancer cells in vivo. Endocrinology.

[68] Wang Z., Zhang X., Shen P., Loggie B.W., Chang Y., Deuel T.F. (2006). A variant of estrogen receptor-α, hER-α36: Transduction of estrogen- and antiestrogen-dependent membrane-initiated mitogenic signaling. Proc. Natl. Acad. Sci. USA.

[69] Revankar C.M., Cimino D.F., Sklar L.A., Arterburn J.B., Prossnitz E.R. (2005). A transmembrane intracellular estrogen receptor mediates rapid cell signaling. Science.

[70] Caulin-Glaser T., Garcia-Cardena G., Sarrel P., Sessa W.C., Bender J.R. (1997). 17 beta-estradiol regulation of human endothelial cell basal nitric oxide release, independent of cytosolic Ca^2+^ mobilization. Circ. Res..

[71] Levin E.R. (2002). Cellular functions of plasma membrane estrogen receptors. Steroids.

[72] Razandi M., Pedram A., Park S.T., Levin E.R. (2003). Proximal events in signaling by plasma membrane estrogen receptors. J. Biol. Chem..

[73] Hall J.M., Couse J.F., Korach K.S. (2001). The multifaceted mechanisms of estradiol and estrogen receptor signaling. J. Biol. Chem..

[74] Prossnitz E.R., Barton M. (2011). The G-protein-coupled estrogen receptor GPER in health and disease. Nat. Rev. Endocrinol..

[75] Bologa C.G., Revankar C.M., Young S.M., Edwards B.S., Arterburn J.B., Kiselyov A.S., Parker M.A., Tkachenko S.E., Savchuck N.P., Sklar L.A. (2006). Virtual and biomolecular screening converge on a selective agonist for GPR30. Nat. Chem. Biol..

[76] Filardo E.J., Quinn J.A., Frackelton A.R., Bland K.I. (2002). Estrogen action via the G protein-coupled receptor, GPR30: Stimulation of adenylyl cyclase and cAMP-mediated attenuation of the epidermal growth factor receptor-to-MAPK signaling axis. Mol. Endocrinol..

[77] Prossnitz E.R., Maggiolini M. (2009). Mechanisms of estrogen signaling and gene expression via GPR30. Mol. Cell. Endocrinol..

[78] Lappano R., De Marco P., De Francesco E.M., Chimento A., Pezzi V., Maggiolini M. (2013). Cross-talk between GPER and growth factor signaling. J. Steroid Biochem. Mol..

[79] Ariazi E.A., Brailoiu E., Yerrum S., Shupp H.A., Slifker M.J., Cunliffe H.E., Black M.A., Donato A.L., Arterburn J.B., Oprea T.I. (2010). The G protein-coupled receptor GPR30 inhibits proliferation of estrogen receptor-positive breast cancer cells. Cancer Res..

[80] Hsu L.H., Chu N.M., Lin Y.F., Kao S.H. (2019). G-Protein Coupled Estrogen Receptor in Breast Cancer. Int. J. Mol. Sci..

[81] Yang H.C., Wang C.Y., Liao H.Q., Wang Q. (2021). Activation of GPER by E2 promotes proliferation, invasion and migration of breast cancer cells by regulating the miR-124/CD151 pathway. Oncol. Lett..

[82] He Y.Y., Cai B., Yang Y.X., Liu X.L., Wan X.P. (2009). Estrogenic G protein-coupled receptor 30 signaling is involved in regulation of endometrial carcinoma by promoting proliferation, invasion potential, and interleukin-6 secretion via the MEK/ERK mitogen-activated protein kinase pathway. Cancer Sci..

[83] Liu H.D., Yan Y., Wen H.X., Jiang X.L., Cao X.F., Zhang G.M., Liu G.Y. (2014). A novel estrogen receptor GPER mediates proliferation induced by 17 beta-estradiol and selective GPER agonist G-1 in estrogen receptor alpha (ER alpha)-negative ovarian cancer cells. Cell Biol. Int..

[84] Chan Q.K., Lam H.M., Ng C.F., Lee A.Y., Chan E.S., Ng H.K., Ho S.M., Lau K.M. (2010). Activation of GPR30 inhibits the growth of prostate cancer cells through sustained activation of Erk1/2, c-jun/c-fos-dependent upregulation of p21, and induction of G(2) cell-cycle arrest. Cell Death Diff..

[85] Wang C., Lv X., He C., Hua G., Tsai M.Y., Davis J.S. (2013). The G-protein-coupled estrogen receptor agonist G-1 suppresses proliferation of ovarian cancer cells by blocking tubulin polymerization. Cell Death Dis..

[86] Pearce S.T., Jordan V.C. (2004). The biological role of estrogen receptors alpha and beta in cancer. Crit. Rev. Oncol. Hematol..

[87] Matsumura S., Ohta T., Yamanouchi K., Liu Z., Sudo T., Kojimahara T., Seino M., Narumi M., Tsutsumi S., Takahashi T. (2017). Activation of estrogen receptor alpha by estradiol and cisplatin induces platinum-resistance in ovarian cancer cells. Cancer Biol. Ther..

[88] Ali S., Coombes R.C. (2000). Estrogen receptor alpha in human breast cancer: Occurrence and significance. J. Mammary Gland Biol. Neoplasia.

[89] Tian W., Teng F., Gao J., Gao C., Liu G., Zhang Y., Yu S., Zhang W., Wang Y., Xue F. (2019). Estrogen and insulin synergistically promote endometrial cancer progression via crosstalk between their receptor signaling pathways. Cancer Biol. Med..

[90] Bonkhoff H. (2018). Estrogen receptor signaling in prostate cancer: Implications for carcinogenesis and tumor progression. Prostate.

[91] Shen Z., Luo H., Li S., Sheng B., Zhao M., Zhu H., Zhu X. (2017). Correlation between estrogen receptor expression and prognosis in epithelial ovarian cancer: A meta-analysis. Oncotarget.

[92] Bocchinfuso W.P., Hively W.P., Couse J.F., Varmus H.E., Korach K.S. (1999). A mouse mammary tumor virus-Wnt-1 transgene induces mammary gland hyperplasia and tumorigenesis in mice lacking estrogen receptor-alpha. Cancer Res..

[93] Ricke W.A., McPherson S.J., Bianco J.J., Cunha G.R., Wang Y., Risbridger G.P. (2008). Prostatic hormonal carcinogenesis is mediated by in situ estrogen production and estrogen receptor alpha signaling. FASEB J..

[94] Di Zazzo E., Galasso G., Giovannelli P., Di Donato M., Bilancio A., Perillo B., Sinisi A.A., Migliaccio A., Castoria G. (2019). Estrogen Receptors in Epithelial-Mesenchymal Transition of Prostate Cancer. Cancers.

[95] Liang Z., Cao J., Tian L., Shen Y., Yang X., Lin Q., Zhang R., Liu H., Du X., Shi J. (2019). Aromatase-induced endogenous estrogen promotes tumour metastasis through estrogen receptor-alpha/matrix metalloproteinase 12 axis activation in castration-resistant prostate cancer. Cancer Lett..

[96] Yu Z., Gao W., Jiang E., Lu F., Zhang L., Shi Z., Wang X., Chen L., Lv T. (2013). Interaction between IGF-IR and ER induced by E2 and IGF-I. PLoS ONE.

[97] du Rusquec P., Blonz C., Frenel J.S., Campone M. (2020). Targeting the PI3K/Akt/mTOR pathway in estrogen-receptor positive HER2 negative advanced breast cancer. Ther. Adv. Med. Oncol..

[98] So C.L., Saunus J.M., Roberts-Thomson S.J., Monteith G.R. (2019). Calcium signalling and breast cancer. Semin. Cell Dev. Biol..

[99] Bouris P., Skandalis S.S., Piperigkou Z., Afratis N., Karamanou K., Aletras A.J., Moustakas A., Theocharis A.D., Karamanos N.K. (2015). Estrogen receptor alpha mediates epithelial to mesenchymal transition, expression of specific matrix effectors and functional properties of breast cancer cells. Matrix Biol..

[100] Diaz Bessone M.I., Gattas M.J., Laporte T., Tanaka M., Simian M. (2019). The Tumor Microenvironment as a Regulator of Endocrine Resistance in Breast Cancer. Front. Endocrinol..

[101] Pagano M.T., Ortona E., Dupuis M.L. (2020). A Role for Estrogen Receptor alpha36 in Cancer Progression. Front. Endocrinol..

[102] Su X., Xu X., Li G., Lin B., Cao J., Teng L. (2014). ER-alpha36: A novel biomarker and potential therapeutic target in breast cancer. Onco Targets Ther..

[103] Thiebaut C., Konan H.P., Guerquin M.J., Chesnel A., Livera G., Le Romancer M., Dumond H. (2020). The Role of ERalpha36 in Development and Tumor Malignancy. Int. J. Mol. Sci..

[104] Tong J.S., Zhang Q.H., Wang Z.B., Li S., Yang C.R., Fu X.Q., Hou Y., Wang Z.Y., Sheng J., Sun Q.Y. (2010). ER-alpha36, a novel variant of ER-alpha, mediates estrogen-stimulated proliferation of endometrial carcinoma cells via the PKCdelta/ERK pathway. PLoS ONE.

[105] Omarjee S., Jacquemetton J., Poulard C., Rochel N., Dejaegere A., Chebaro Y., Treilleux I., Marangoni E., Corbo L., Romancer M.L. (2017). The molecular mechanisms underlying the ERalpha-36-mediated signaling in breast cancer. Oncogene.

[106] Chaudhri R.A., Olivares-Navarrete R., Cuenca N., Hadadi A., Boyan B.D., Schwartz Z. (2012). Membrane estrogen signaling enhances tumorigenesis and metastatic potential of breast cancer cells via estrogen receptor-alpha36 (ERalpha36). J. Biol. Chem..

[107] Wang Z.Y., Yin L. (2015). Estrogen receptor alpha-36 (ER-alpha36): A new player in human breast cancer. Mol. Cell. Endocrinol..

[108] Zou Y., Ding L., Coleman M., Wang Z. (2009). Estrogen receptor-alpha (ER-alpha) suppresses expression of its variant ER-alpha 36. FEBS Lett..

[109] Kang L., Zhang X., Xie Y., Tu Y., Wang D., Liu Z., Wang Z.Y. (2010). Involvement of estrogen receptor variant ER-alpha36, not GPR30, in nongenomic estrogen signaling. Mol. Endocrinol..

[110] Zhang X., Ding L., Kang L., Wang Z.Y. (2012). Estrogen receptor-alpha 36 mediates mitogenic antiestrogen signaling in ER-negative breast cancer cells. PLoS ONE.

[111] Guillette T.C., Jackson T.W., Belcher S.M. (2018). Duality of estrogen receptor beta action in cancer progression. Curr. Opin. Pharmacol..

[112] Di Zazzo E., Galasso G., Giovannelli P., Di Donato M., Castoria G. (2018). Estrogens and Their Receptors in Prostate Cancer: Therapeutic Implications. Front. Oncol..

[113] Liu M.M., Albanese C., Anderson C.M., Hilty K., Webb P., Uht R.M., Price R.H., Pestell R.G., Kushner P.J. (2002). Opposing action of estrogen receptors alpha and beta on cyclin D1 gene expression. J. Biol. Chem..

[114] Williams C., Edvardsson K., Lewandowski S.A., Strom A., Gustafsson J.A. (2008). A genome-wide study of the repressive effects of estrogen receptor beta on estrogen receptor alpha signaling in breast cancer cells. Oncogene.

[115] Mishra A.K., Abrahamsson A., Dabrosin C. (2016). Fulvestrant inhibits growth of triple negative breast cancer and synergizes with tamoxifen in ERalpha positive breast cancer by up-regulation of ERbeta. Oncotarget.

[116] Ma R., Karthik G.M., Lovrot J., Haglund F., Rosin G., Katchy A., Zhang X., Viberg L., Frisell J., Williams C. (2017). Estrogen Receptor beta as a Therapeutic Target in Breast Cancer Stem Cells. J. Natl. Cancer Inst..

[117] Liu J., Guo H., Mao K., Zhang K., Deng H., Liu Q. (2016). Impact of estrogen receptor-beta expression on breast cancer prognosis: A meta-analysis. Breast Cancer Res. Treat..

[118] Bialesova L., Xu L., Gustafsson J.A., Haldosen L.A., Zhao C., Dahlman-Wright K. (2017). Estrogen receptor beta2 induces proliferation and invasiveness of triple negative breast cancer cells: Association with regulation of PHD3 and HIF-1alpha. Oncotarget.

[119] Ellem S.J., Risbridger G.P. (2007). Treating prostate cancer: A rationale for targeting local oestrogens. Nat. Rev. Cancer.

[120] Dey P., Strom A., Gustafsson J.A. (2014). Estrogen receptor beta upregulates FOXO3a and causes induction of apoptosis through PUMA in prostate cancer. Oncogene.

[121] Wu W.F., Maneix L., Insunza J., Nalvarte I., Antonson P., Kere J., Yu N.Y., Tohonen V., Katayama S., Einarsdottir E. (2017). Estrogen receptor beta, a regulator of androgen receptor signaling in the mouse ventral prostate. Proc. Natl. Acad. Sci. USA.

[122] Fujimura T., Takahashi S., Urano T., Ogawa S., Ouchi Y., Kitamura T., Muramatsu M., Inoue S. (2001). Differential expression of estrogen receptor beta (ERbeta) and its C-terminal truncated splice variant ERbetacx as prognostic predictors in human prostatic cancer. Biochem. Biophys. Res. Commun..

[123] Leung Y.K., Lam H.M., Wu S., Song D., Levin L., Cheng L., Wu C.L., Ho S.M. (2010). Estrogen receptor beta2 and beta5 are associated with poor prognosis in prostate cancer, and promote cancer cell migration and invasion. Endocr. Relat. Cancer.

[124] Lewis-Wambi J.S., Jordan V.C. (2009). Estrogen regulation of apoptosis: How can one hormone stimulate and inhibit?. Breast Cancer Res..

[125] Sweeney E.E., Fan P., Jordan V.C. (2014). Mechanisms underlying differential response to estrogen-induced apoptosis in long-term estrogen-deprived breast cancer cells. Int. J. Oncol..

[126] Escher T.E., Lui A.J., Geanes E.S., Walter K.R., Tawfik O., Hagan C.R., Lewis-Wambi J. (2019). Interaction between MUC1 and STAT1 Drives IFITM1 Overexpression in Aromatase Inhibitor-Resistant Breast Cancer Cells and Mediates Estrogen-Induced Apoptosis. Mol. Cancer Res. MCR.

[127] Sengupta S., Sevigny C.M., Bhattacharya P., Jordan V.C., Clarke R. (2019). Estrogen-Induced Apoptosis in Breast Cancers Is Phenocopied by Blocking Dephosphorylation of Eukaryotic Initiation Factor 2 Alpha (eIF2alpha) Protein. Mol. Cancer Res..

[128] Maximov P.Y., Abderrahman B., Curpan R.F., Hawsawi Y.M., Fan P., Jordan V.C. (2018). A unifying biology of sex steroid-induced apoptosis in prostate and breast cancers. Endocr. Relat. Cancer.

[129] Harbeck N., Penault-Llorca F., Cortes J., Gnant M., Houssami N., Poortmans P., Ruddy K., Tsang J., Cardoso F. (2019). Breast cancer. Nat. Rev. Dis. Primers.

[130] Johnson K.S., Conant E.F., Soo M.S. (2021). Molecular Subtypes of Breast Cancer: A Review for Breast Radiologists. J. Breast Imaging.

[131] Lima Z.S., Ghadamzadeh M., Arashloo F.T., Amjad G., Ebadi M.R., Younesi L. (2019). Recent advances of therapeutic targets based on the molecular signature in breast cancer: Genetic mutations and implications for current treatment paradigms. J. Hematol. Oncol..

[132] Montalto F.I., De Amicis F. (2020). Cyclin D1 in Cancer: A Molecular Connection for Cell Cycle Control, Adhesion and Invasion in Tumor and Stroma. Cells.

[133] Travis R.C., Key T.J. (2003). Oestrogen exposure and breast cancer risk. Breast Cancer Res..

[134] Lu W., Katzenellenbogen B.S. (2017). Estrogen Receptor-beta Modulation of the ERalpha-p53 Loop Regulating Gene Expression, Proliferation, and Apoptosis in Breast Cancer. Horm. Cancer.

[135] Bocchinfuso W.P., Lindzey J.K., Hewitt S.C., Clark J.A., Myers P.H., Cooper R., Korach K.S. (2000). Induction of mammary gland development in estrogen receptor-alpha knockout mice. Endocrinology.

[136] Krege J.H., Hodgin J.B., Couse J.F., Enmark E., Warner M., Mahler J.F., Sar M., Korach K.S., Gustafsson J.A., Smithies O. (1998). Generation and reproductive phenotypes of mice lacking estrogen receptor beta. Proc. Natl. Acad. Sci. USA.

[137] Kurebayashi J., Otsuki T., Kunisue H., Tanaka K., Yamamoto S., Sonoo H. (2000). Expression levels of estrogen receptor-alpha, estrogen receptor-beta, coactivators, and corepressors in breast cancer. Clin. Cancer Res..

[138] Tan H., Zhong Y., Pan Z. (2009). Autocrine regulation of cell proliferation by estrogen receptor-alpha in estrogen receptor-alpha-positive breast cancer cell lines. BMC Cancer.

[139] Hou Y.F., Yuan S.T., Li H.C., Wu J., Lu J.S., Liu G., Lu L.J., Shen Z.Z., Ding J., Shao Z.M. (2004). ERbeta exerts multiple stimulative effects on human breast carcinoma cells. Oncogene.

[140] De Amicis F., Guido C., Santoro M., Lanzino M., Panza S., Avena P., Panno M.L., Perrotta I., Aquila S., Ando S. (2014). A novel functional interplay between Progesterone Receptor-B and PTEN, via AKT, modulates autophagy in breast cancer cells. J. Cell. Mol. Med..

[141] Daniel A.R., Hagan C.R., Lange C.A. (2011). Progesterone receptor action: Defining a role in breast cancer. Expert Rev. Endocrinol. Metab..

[142] Waks A.G., Winer E.P. (2019). Breast Cancer Treatment: A Review. JAMA.

[143] Soto A.M., Sonnenschein C. (2001). The two faces of janus: Sex steroids as mediators of both cell proliferation and cell death. J. Natl. Cancer Inst..

[144] Santen R.J. (2007). The oestrogen paradox: A hypothesis. Endokrynol Pol..

[145] Coelingh Bennink H.J., Verhoeven C., Dutman A.E., Thijssen J. (2017). The use of high-dose estrogens for the treatment of breast cancer. Maturitas.

[146] Song R.X., Mor G., Naftolin F., McPherson R.A., Song J., Zhang Z., Yue W., Wang J., Santen R.J. (2001). Effect of long-term estrogen deprivation on apoptotic responses of breast cancer cells to 17beta-estradiol. J. Natl. Cancer Inst..

[147] Masamura S., Santner S.J., Heitjan D.F., Santen R.J. (1995). Estrogen deprivation causes estradiol hypersensitivity in human breast cancer cells. J. Clin. Endocrinol. Metab..

[148] Suba Z. (2015). The pitfall of the transient, inconsistent anticancer capacity of antiestrogens and the mechanism of apparent antiestrogen resistance. Drug Des. Devel. Ther..

[149] Sonnenschein C., Szelei J., Nye T.L., Soto A.M. (1994). Control of cell proliferation of human breast MCF7 cells; serum and estrogen resistant variants. Oncol. Res..

[150] Jiang S.Y., Wolf D.M., Yingling J.M., Chang C., Jordan V.C. (1992). An estrogen receptor positive MCF-7 clone that is resistant to anti-estrogens and estradiol. Mol. Cell. Endocrinol..

[151] Wolf D.M., Jordan V.C. (1993). A laboratory model to explain the survival advantage observed in patients taking adjuvant tamoxifen therapy. Recent Results Cancer Res..

[152] Yao K., Lee E.S., Bentrem D.J., England G., Schafer J.I., O’Regan R.M., Jordan V.C. (2000). Antitumor action of physiological estradiol on tamoxifen-stimulated breast tumors grown in athymic mice. Clin. Cancer Res..

[153] Liu H., Lee E.S., Gajdos C., Pearce S.T., Chen B., Osipo C., Loweth J., McKian K., De Los Reyes A., Wing L. (2003). Apoptotic action of 17beta-estradiol in raloxifene-resistant MCF-7 cells in vitro and in vivo. J. Natl. Cancer Inst..

[154] Osipo C., Gajdos C., Liu H., Chen B., Jordan V.C. (2003). Paradoxical action of fulvestrant in estradiol-induced regression of tamoxifen-stimulated breast cancer. J. Natl. Cancer Inst..

[155] Jordan V.C. (2015). The new biology of estrogen-induced apoptosis applied to treat and prevent breast cancer. Endocr. Relat. Cancer.

[156] Hosford S.R., Shee K., Wells J.D., Traphagen N.A., Fields J.L., Hampsch R.A., Kettenbach A.N., Demidenko E., Miller T.W. (2019). Estrogen therapy induces an unfolded protein response to drive cell death in ER+ breast cancer. Mol. Oncol..

[157] Song R.X., Zhang Z., Mor G., Santen R.J. (2005). Down-regulation of Bcl-2 enhances estrogen apoptotic action in long-term estradiol-depleted ER(+) breast cancer cells. Apoptosis.

[158] Szelei J., Soto A.M., Geck P., Desronvil M., Prechtl N.V., Weill B.C., Sonnenschein C. (2000). Identification of human estrogen-inducible transcripts that potentially mediate the apoptotic response in breast cancer. J. Steroid Biochem. Mol..

[159] Lewis J.S., Meeke K., Osipo C., Ross E.A., Kidawi N., Li T., Bell E., Chandel N.S., Jordan V.C. (2005). Intrinsic mechanism of estradiol-induced apoptosis in breast cancer cells resistant to estrogen deprivation. J. Natl. Cancer Inst..

[160] Ariazi E.A., Cunliffe H.E., Lewis-Wambi J.S., Slifker M.J., Willis A.L., Ramos P., Tapia C., Kim H.R., Yerrum S., Sharma C.G. (2011). Estrogen induces apoptosis in estrogen deprivation-resistant breast cancer through stress responses as identified by global gene expression across time. Proc. Natl. Acad. Sci. USA.

[161] Lui A.J., Geanes E.S., Ogony J., Behbod F., Marquess J., Valdez K., Jewell W., Tawfik O., Lewis-Wambi J. (2017). IFITM1 suppression blocks proliferation and invasion of aromatase inhibitor-resistant breast cancer in vivo by JAK/STAT-mediated induction of p21. Cancer Lett..

[162] Fan P., Griffith O.L., Agboke F.A., Anur P., Zou X., McDaniel R.E., Creswell K., Kim S.H., Katzenellenbogen J.A., Gray J.W. (2013). c-Src modulates estrogen-induced stress and apoptosis in estrogen-deprived breast cancer cells. Cancer Res.

[163] Fan P., Tyagi A.K., Agboke F.A., Mathur R., Pokharel N., Jordan V.C. (2018). Modulation of nuclear factor-kappa B activation by the endoplasmic reticulum stress sensor PERK to mediate estrogen-induced apoptosis in breast cancer cells. Cell Death Discov..

[164] Walter P., Ron D. (2011). The unfolded protein response: From stress pathway to homeostatic regulation. Science.

[165] Tabas I., Ron D. (2011). Integrating the mechanisms of apoptosis induced by endoplasmic reticulum stress. Nature Cell Biol..

[166] Yde C.W., Emdal K.B., Guerra B., Lykkesfeldt A.E. (2012). NFkappaB signaling is important for growth of antiestrogen resistant breast cancer cells. Breast Cancer Res. Treat..

[167] Moreno J.A., Radford H., Peretti D., Steinert J.R., Verity N., Martin M.G., Halliday M., Morgan J., Dinsdale D., Ortori C.A. (2012). Sustained translational repression by eIF2alpha-P mediates prion neurodegeneration. Nature.

[168] Novoa I., Zeng H., Harding H.P., Ron D. (2001). Feedback inhibition of the unfolded protein response by GADD34-mediated dephosphorylation of eIF2alpha. J. Cell Biol..

[169] Brush M.H., Weiser D.C., Shenolikar S. (2003). Growth arrest and DNA damage-inducible protein GADD34 targets protein phosphatase 1 alpha to the endoplasmic reticulum and promotes dephosphorylation of the alpha subunit of eukaryotic translation initiation factor 2. Mol. Cell. Biol..

[170] Jousse C., Oyadomari S., Novoa I., Lu P., Zhang Y., Harding H.P., Ron D. (2003). Inhibition of a constitutive translation initiation factor 2alpha phosphatase, CReP, promotes survival of stressed cells. J. Cell Biol..

[171] Siegel R.L., Miller K.D., Jemal A. (2020). Cancer statistics, 2020. CA Cancer J. Clin..

[172] Barsouk A., Padala S.A., Vakiti A., Mohammed A., Saginala K., Thandra K.C., Rawla P., Barsouk A. (2020). Epidemiology, Staging and Management of Prostate Cancer. Med. Sci..

[173] Cimadamore A., Mazzucchelli R., Lopez-Beltran A., Massari F., Santoni M., Scarpelli M., Cheng L., Montironi R. (2021). Prostate Cancer in 2021: Novelties in Prognostic and Therapeutic Biomarker Evaluation. Cancers.

[174] Paschalis A., de Bono J.S. (2020). Prostate Cancer 2020: "The Times They Are a’Changing". Cancer Cell.

[175] Westaby D., Maza M., Paschalis A., Jimenez-Vacas J.M., Welti J., de Bono J., Sharp A. (2021). A New Old Target: Androgen Receptor Signaling and Advanced Prostate Cancer. Annu. Rev. Pharmacol. Toxicol..

[176] Fujimura T., Takayama K., Takahashi S., Inoue S. (2018). Estrogen and Androgen Blockade for Advanced Prostate Cancer in the Era of Precision Medicine. Cancers.

[177] Huggins C., Hodges C.V. (1972). Studies on prostatic cancer. I. The effect of castration, of estrogen and androgen injection on serum phosphatases in metastatic carcinoma of the prostate. CA Cancer J. Clin..

[178] Koutsilieris M., Tolis G. (1985). Long-term follow-up of patients with advanced prostatic carcinoma treated with either buserelin (HOE 766) or orchiectomy: Classification of variables associated with disease outcome. Prostate.

[179] Klotz L., McNeill I., Fleshner N. (1999). A phase 1–2 trial of diethylstilbestrol plus low dose warfarin in advanced prostate carcinoma. J. Urol..

[180] Ho S.M., Leung Y.K., Chung I. (2006). Estrogens and antiestrogens as etiological factors and therapeutics for prostate cancer. Ann. N. Y. Acad..

[181] Aurilio G., Cimadamore A., Mazzucchelli R., Lopez-Beltran A., Verri E., Scarpelli M., Massari F., Cheng L., Santoni M., Montironi R. (2020). Androgen Receptor Signaling Pathway in Prostate Cancer: From Genetics to Clinical Applications. Cells.

[182] Royuela M., de Miguel M.P., Bethencourt F.R., Sanchez-Chapado M., Fraile B., Arenas M.I., Paniagua R. (2001). Estrogen receptors alpha and beta in the normal, hyperplastic and carcinomatous human prostate. J. Endocrinol..

[183] Takizawa I., Lawrence M.G., Balanathan P., Rebello R., Pearson H.B., Garg E., Pedersen J., Pouliot N., Nadon R., Watt M.J. (2015). Estrogen receptor alpha drives proliferation in PTEN-deficient prostate carcinoma by stimulating survival signaling, MYC expression and altering glucose sensitivity. Oncotarget.

[184] Fixemer T., Remberger K., Bonkhoff H. (2003). Differential expression of the estrogen receptor beta (ERbeta) in human prostate tissue, premalignant changes, and in primary, metastatic, and recurrent prostatic adenocarcinoma. Prostate.

[185] Gehrig J., Kaulfuss S., Jarry H., Bremmer F., Stettner M., Burfeind P., Thelen P. (2017). Prospects of estrogen receptor beta activation in the treatment of castration-resistant prostate cancer. Oncotarget.

[186] Christoforou P., Christopoulos P.F., Koutsilieris M. (2014). The role of estrogen receptor beta in prostate cancer. Mol. Med..

[187] McPherson S.J., Hussain S., Balanathan P., Hedwards S.L., Niranjan B., Grant M., Chandrasiri U.P., Toivanen R., Wang Y., Taylor R.A. (2010). Estrogen receptor-beta activated apoptosis in benign hyperplasia and cancer of the prostate is androgen independent and TNFalpha mediated. Proc. Natl. Acad. Sci. USA.

[188] Yun H., Xie J., Olumi A.F., Ghosh R., Kumar A.P. (2015). Activation of AKR1C1/ERbeta induces apoptosis by downregulation of c-FLIP in prostate cancer cells: A prospective therapeutic opportunity. Oncotarget.

[189] Xiao L., Xiao M., Zou M., Xu W. (2017). Estrogen receptor beta inhibits prostate cancer cell proliferation through downregulating TGF-beta1/IGF-1 signaling. Int. J. Clin. Exp. Pathol..

[190] Xiao L., Luo Y., Tai R., Zhang N. (2019). Estrogen receptor beta suppresses inflammation and the progression of prostate cancer. Mol. Med. Rep..

[191] Treeck O., Pfeiler G., Mitter D., Lattrich C., Piendl G., Ortmann O. (2007). Estrogen receptor {beta}1 exerts antitumoral effects on SK-OV-3 ovarian cancer cells. J. Endocrinol..

[192] Wu J., Miao C., Lv X., Zhang Y., Li Y., Wang D. (2019). Estrogen regulates forkhead transcription factor 2 to promote apoptosis of human ovarian granulosa-like tumor cells. J. Steroid Biochem. Mol..

[193] Treeck O., Diepolder E., Skrzypczak M., Schuler-Toprak S., Ortmann O. (2019). Knockdown of estrogen receptor beta increases proliferation and affects the transcriptome of endometrial adenocarcinoma cells. BMC Cancer.

[194] McCourt C., Maxwell P., Mazzucchelli R., Montironi R., Scarpelli M., Salto-Tellez M., O’Sullivan J.M., Longley D.B., Waugh D.J. (2012). Elevation of c-FLIP in castrate-resistant prostate cancer antagonizes therapeutic response to androgen receptor-targeted therapy. Clin. Cancer Res..

[195] Arora T., Mullangi S., Lekkala M.R. (2022). Ovarian Cancer.

[196] De Leo A., Santini D., Ceccarelli C., Santandrea G., Palicelli A., Acquaviva G., Chiarucci F., Rosini F., Ravegnini G., Pession A. (2021). What Is New on Ovarian Carcinoma: Integrated Morphologic and Molecular Analysis Following the New 2020 World Health Organization Classification of Female Genital Tumors. Diagnostics.

[197] Momenimovahed Z., Tiznobaik A., Taheri S., Salehiniya H. (2019). Ovarian cancer in the world: Epidemiology and risk factors. Int. J. Women’s Health.

[198] Torre L.A., Trabert B., DeSantis C.E., Miller K.D., Samimi G., Runowicz C.D., Gaudet M.M., Jemal A., Siegel R.L. (2018). Ovarian cancer statistics, 2018. CA Cancer J. Clin..

[199] Reid B.M., Permuth J.B., Sellers T.A. (2017). Epidemiology of ovarian cancer: A review. Cancer Biol. Med..

[200] Mungenast F., Thalhammer T. (2014). Estrogen biosynthesis and action in ovarian cancer. Front. Endocrinol..

[201] Bossard C., Busson M., Vindrieux D., Gaudin F., Machelon V., Brigitte M., Jacquard C., Pillon A., Balaguer P., Balabanian K. (2012). Potential role of estrogen receptor beta as a tumor suppressor of epithelial ovarian cancer. PLoS ONE.

[202] Bardin A., Hoffmann P., Boulle N., Katsaros D., Vignon F., Pujol P., Lazennec G. (2004). Involvement of estrogen receptor beta in ovarian carcinogenesis. Cancer Res..

[203] Rutherford T., Brown W.D., Sapi E., Aschkenazi S., Munoz A., Mor G. (2000). Absence of estrogen receptor-beta expression in metastatic ovarian cancer. Obstet. Gynecol..

[204] Schuler-Toprak S., Weber F., Skrzypczak M., Ortmann O., Treeck O. (2018). Estrogen receptor beta is associated with expression of cancer associated genes and survival in ovarian cancer. BMC Cancer.

[205] Suzuki F., Akahira J., Miura I., Suzuki T., Ito K., Hayashi S., Sasano H., Yaegashi N. (2008). Loss of estrogen receptor beta isoform expression and its correlation with aberrant DNA methylation of the 5’-untranslated region in human epithelial ovarian carcinoma. Cancer Sci..

[206] Skvortsova K., Stirzaker C., Taberlay P. (2019). The DNA methylation landscape in cancer. Essays Biochem..

[207] Schuler-Toprak S., Moehle C., Skrzypczak M., Ortmann O., Treeck O. (2017). Effect of estrogen receptor beta agonists on proliferation and gene expression of ovarian cancer cells. BMC Cancer.

[208] Kim J.H., Yoon S., Park M., Park H.O., Ko J.J., Lee K., Bae J. (2011). Differential apoptotic activities of wild-type FOXL2 and the adult-type granulosa cell tumor-associated mutant FOXL2 (C134W). Oncogene.

[209] Georges A., L’Hote D., Todeschini A.L., Auguste A., Legois B., Zider A., Veitia R.A. (2014). The transcription factor FOXL2 mobilizes estrogen signaling to maintain the identity of ovarian granulosa cells. eLife.

[210] Bruggmann D., Ouassou K., Klingelhofer D., Bohlmann M.K., Jaque J., Groneberg D.A. (2020). Endometrial cancer: Mapping the global landscape of research. J. Transl. Med..

[211] Lu K.H., Broaddus R.R. (2020). Endometrial Cancer. N. Engl. J. Med..

[212] Urick M.E., Bell D.W. (2019). Clinical actionability of molecular targets in endometrial cancer. Nat. Rev. Cancer.

[213] Zaino R.J., Kurman R.J., Diana K.L., Morrow C.P. (1995). The utility of the revised International Federation of Gynecology and Obstetrics histologic grading of endometrial adenocarcinoma using a defined nuclear grading system. A Gynecologic Oncology Group study. Cancer.

[214] Shen F., Gao Y., Ding J., Chen Q. (2017). Is the positivity of estrogen receptor or progesterone receptor different between type 1 and type 2 endometrial cancer?. Oncotarget.

[215] Rodriguez A.C., Blanchard Z., Maurer K.A., Gertz J. (2019). Estrogen Signaling in Endometrial Cancer: A Key Oncogenic Pathway with Several Open Questions. Horm. Cancer.

[216] Gao Y., Zhao M., Dai X., Tong M., Wei J., Chen Q. (2016). The prevalence of endometrial cancer in pre- and postmenopausal Chinese women. Menopause.

[217] Setiawan V.W., Yang H.P., Pike M.C., McCann S.E., Yu H., Xiang Y.B., Wolk A., Wentzensen N., Weiss N.S., Webb P.M. (2013). Type I and II endometrial cancers: Have they different risk factors?. J. Clin. Oncol..

[218] Srijaipracharoen S., Tangjitgamol S., Tanvanich S., Manusirivithaya S., Khunnarong J., Thavaramara T., Leelahakorn S., Pataradool K. (2010). Expression of ER, PR, and Her-2/neu in endometrial cancer: A clinicopathological study. Asian Pac. J. Cancer Prev..

[219] Dai D., Wolf D.M., Litman E.S., White M.J., Leslie K.K. (2002). Progesterone inhibits human endometrial cancer cell growth and invasiveness: Down-regulation of cellular adhesion molecules through progesterone B receptors. Cancer Res..

[220] Concin N., Matias-Guiu X., Vergote I., Cibula D., Mirza M.R., Marnitz S., Ledermann J., Bosse T., Chargari C., Fagotti A. (2021). ESGO/ESTRO/ESP guidelines for the management of patients with endometrial carcinoma. Int. J. Gynecol. Cancer..

[221] Brandenberger A.W., Lebovic D.I., Tee M.K., Ryan I.P., Tseng J.F., Jaffe R.B., Taylor R.N. (1999). Oestrogen receptor (ER)-alpha and ER-beta isoforms in normal endometrial and endometriosis-derived stromal cells. Mol. Hum. Reprod..

[222] Mylonas I., Jeschke U., Shabani N., Kuhn C., Balle A., Kriegel S., Kupka M.S., Friese K. (2004). Immunohistochemical analysis of estrogen receptor alpha, estrogen receptor beta and progesterone receptor in normal human endometrium. Acta Histochem..

[223] Utsunomiya H., Suzuki T., Harada N., Ito K., Matsuzaki S., Konno R., Sato S., Yajima A., Sasano H. (2000). Analysis of estrogen receptor alpha and beta in endometrial carcinomas: Correlation with ER beta and clinicopathologic findings in 45 cases. Int. J. Gynecol. Pathol..

[224] Haring J., Skrzypczak M., Stegerer A., Lattrich C., Weber F., Gorse R., Ortmann O., Treeck O. (2012). Estrogen receptor beta transcript variants associate with oncogene expression in endometrial cancer. Int. J. Mol. Med..

[225] Skrzypczak M., Bieche I., Szymczak S., Tozlu S., Lewandowski S., Girault I., Radwanska K., Szczylik C., Jakowicki J.A., Lidereau R. (2004). Evaluation of mRNA expression of estrogen receptor beta and its isoforms in human normal and neoplastic endometrium. Int. J. Cancer.

[226] Leygue E., Dotzlaw H., Watson P.H., Murphy L.C. (1999). Expression of estrogen receptor beta1, beta2, and beta5 messenger RNAs in human breast tissue. Cancer Res..

[227] Smuc T., Rizner T.L. (2009). Aberrant pre-receptor regulation of estrogen and progesterone action in endometrial cancer. Mol. Cell. Endocrinol..

[228] Chakravarty D., Srinivasan R., Ghosh S., Gopalan S., Rajwanshi A., Majumdar S. (2007). Estrogen receptor beta1 and the beta2/betacx isoforms in nonneoplastic endometrium and in endometrioid carcinoma. Int. J. Gynecol. Cancer.

[229] Frontini M., Soutoglou E., Argentini M., Bole-Feysot C., Jost B., Scheer E., Tora L. (2005). TAF9b (formerly TAF9L) is a bona fide TAF that has unique and overlapping roles with TAF9. Mol. Cell. Biol..

[230] Yu F., Bracken C.P., Pillman K.A., Lawrence D.M., Goodall G.J., Callen D.F., Neilsen P.M. (2015). p53 Represses the Oncogenic Sno-MiR-28 Derived from a SnoRNA. PLoS ONE.

[231] Boccellino M., Vanacore D., Zappavigna S., Cavaliere C., Rossetti S., D’Aniello C., Chieffi P., Amler E., Buonerba C., Di Lorenzo G. (2017). Testicular cancer from diagnosis to epigenetic factors. Oncotarget.

[232] Rajpert-De Meyts E. (2006). Developmental model for the pathogenesis of testicular carcinoma in situ: Genetic and environmental aspects. Hum. Reprod. Update.

[233] Chimento A., De Luca A., Nocito M.C., Avena P., La Padula D., Zavaglia L., Pezzi V. (2020). Role of GPER-Mediated Signaling in Testicular Functions and Tumorigenesis. Cells.

[234] Chimento A., Sirianni R., Casaburi I., Pezzi V. (2014). GPER Signaling in Spermatogenesis and Testicular Tumors. Front. Endocrinol..

[235] Carreau S., Chimento A., Bois C., Sirianni R., Delalande C., Pezzi V. (2011). Rapid Estrogen Signaling in Spermatogenesis. Immunol. Endocr. Metab. Agents Med. Chem..

[236] Carreau S., Hess R.A. (2010). Oestrogens and spermatogenesis. Philos. Trans. R. Soc. Lond. B Biol. Sci..

[237] Chimento A., Sirianni R., Zolea F., Bois C., Delalande C., Ando S., Maggiolini M., Aquila S., Carreau S., Pezzi V. (2011). Gper and ESRs are expressed in rat round spermatids and mediate oestrogen-dependent rapid pathways modulating expression of cyclin B1 and Bax. Int. J. Androl..

[238] Chimento A., Sirianni R., Delalande C., Silandre D., Bois C., Ando S., Maggiolini M., Carreau S., Pezzi V. (2010). 17 beta-estradiol activates rapid signaling pathways involved in rat pachytene spermatocytes apoptosis through GPR30 and ER alpha. Mol. Cell. Endocrinol..

[239] Chimento A., Sirianni R., Casaburi I., Ruggiero C., Maggiolini M., Ando S., Pezzi V. (2012). 17beta-Estradiol activates GPER- and ESR1-dependent pathways inducing apoptosis in GC-2 cells, a mouse spermatocyte-derived cell line. Mol. Cell. Endocrinol..

[240] Lucas T.F., Royer C., Siu E.R., Lazari M.F., Porto C.S. (2010). Expression and signaling of G protein-coupled estrogen receptor 1 (GPER) in rat sertoli cells. Biol. Reprod..

[241] Chevalier N., Paul-Bellon R., Camparo P., Michiels J.F., Chevallier D., Fenichel P. (2014). Genetic variants of GPER/GPR30, a novel estrogen-related G protein receptor, are associated with human seminoma. Int. J. Mol. Sci..

[242] Sandner F., Welter H., Schwarzer J.U., Kohn F.M., Urbanski H.F., Mayerhofer A. (2014). Expression of the oestrogen receptor GPER by testicular peritubular cells is linked to sexual maturation and male fertility. Andrology.

[243] Lucas T.F., Pimenta M.T., Pisolato R., Lazari M.F., Porto C.S. (2011). 17beta-estradiol signaling and regulation of Sertoli cell function. Spermatogenesis.

[244] Royer C., Lucas T.F., Lazari M.F., Porto C.S. (2012). 17Beta-estradiol signaling and regulation of proliferation and apoptosis of rat Sertoli cells. Biol. Reprod..

[245] Yang W.R., Zhu F.W., Zhang J.J., Wang Y., Zhang J.H., Lu C., Wang X.Z. (2017). PI3K/Akt Activated by GPR30 and Src Regulates 17beta-Estradiol-Induced Cultured Immature Boar Sertoli Cells Proliferation. Reprod. Sci..

[246] Ge L.C., Chen Z.J., Liu H.Y., Zhang K.S., Liu H., Huang H.B., Zhang G., Wong C.K., Giesy J.P., Du J. (2014). Involvement of activating ERK1/2 through G protein coupled receptor 30 and estrogen receptor alpha/beta in low doses of bisphenol A promoting growth of Sertoli TM4 cells. Toxicol. Lett..

[247] Sirianni R., Chimento A., Ruggiero C., De Luca A., Lappano R., Ando S., Maggiolini M., Pezzi V. (2008). The novel estrogen receptor, G protein-coupled receptor 30, mediates the proliferative effects induced by 17beta-estradiol on mouse spermatogonial GC-1 cell line. Endocrinology.

[248] Sheng Z.G., Zhu B.Z. (2011). Low concentrations of bisphenol A induce mouse spermatogonial cell proliferation by G protein-coupled receptor 30 and estrogen receptor-alpha. Environ. Health Perspect..

[249] Sheng Z.G., Huang W., Liu Y.X., Zhu B.Z. (2013). Bisphenol A at a low concentration boosts mouse spermatogonial cell proliferation by inducing the G protein-coupled receptor 30 expression. Toxicol. Appl. Pharmacol..

[250] Wang C., Zhang J., Li Q., Zhang T., Deng Z., Lian J., Jia D., Li R., Zheng T., Ding X. (2017). Low concentration of BPA induces mice spermatocytes apoptosis via GPR30. Oncotarget.

[251] Milon A., Kaczmarczyk M., Pawlicki P., Bilinska B., Duliban M., Gorowska-Wojtowicz E., Tworzydlo W., Kotula-Balak M. (2019). Do estrogens regulate lipid status in testicular steroidogenic Leydig cell?. Acta Histochem..

[252] Vaucher L., Funaro M.G., Mehta A., Mielnik A., Bolyakov A., Prossnitz E.R., Schlegel P.N., Paduch D.A. (2014). Activation of GPER-1 estradiol receptor downregulates production of testosterone in isolated rat Leydig cells and adult human testis. PLoS ONE.

[253] Pawlicki P., Hejmej A., Milon A., Lustofin K., Plachno B.J., Tworzydlo W., Gorowska-Wojtowicz E., Pawlicka B., Kotula-Balak M., Bilinska B. (2019). Telocytes in the mouse testicular interstitium: Implications of G-protein-coupled estrogen receptor (GPER) and estrogen-related receptor (ERR) in the regulation of mouse testicular interstitial cells. Protoplasma.

[254] Milon A., Pawlicki P., Rak A., Mlyczynska E., Plachno B.J., Tworzydlo W., Gorowska-Wojtowicz E., Bilinska B., Kotula-Balak M. (2019). Telocytes are localized to testis of the bank vole (Myodes glareolus) and are affected by lighting conditions and G-coupled membrane estrogen receptor (GPER) signaling. Gen. Comp. Endocrinol..

[255] Guido C., Panza S., Santoro M., Avena P., Panno M.L., Perrotta I., Giordano F., Casaburi I., Catalano S., De Amicis F. (2012). Estrogen receptor beta (ERbeta) produces autophagy and necroptosis in human seminoma cell line through the binding of the Sp1 on the phosphatase and tensin homolog deleted from chromosome 10 (PTEN) promoter gene. Cell Cycle.

[256] Bouskine A., Nebout M., Mograbi B., Brucker-Davis F., Roger C., Fenichel P. (2008). Estrogens promote human testicular germ cell cancer through a membrane-mediated activation of extracellular regulated kinase and protein kinase A. Endocrinology.

[257] Bouskine A., Nebout M., Brucker-Davis F., Benahmed M., Fenichel P. (2009). Low doses of bisphenol A promote human seminoma cell proliferation by activating PKA and PKG via a membrane G-protein-coupled estrogen receptor. Environ. Health Perspect.

[258] Chevalier N., Bouskine A., Fenichel P. (2012). Bisphenol A promotes testicular seminoma cell proliferation through GPER/GPR30. Int. J. Cancer.

[259] Chevalier N., Vega A., Bouskine A., Siddeek B., Michiels J.F., Chevallier D., Fenichel P. (2012). GPR30, the non-classical membrane G protein related estrogen receptor, is overexpressed in human seminoma and promotes seminoma cell proliferation. PLoS ONE.

[260] Roger C., Lambard S., Bouskine A., Mograbi B., Chevallier D., Nebout M., Pointis G., Carreau S., Fenichel P. (2005). Estrogen-induced growth inhibition of human seminoma cells expressing estrogen receptor beta and aromatase. J. Mol. Endocrinol..

[261] Wallacides A., Chesnel A., Ajj H., Chillet M., Flament S., Dumond H. (2012). Estrogens promote proliferation of the seminoma-like TCam-2 cell line through a GPER-dependent ERalpha36 induction. Mol. Cell. Endocrinol..

[262] Boscia F., Passaro C., Gigantino V., Perdona S., Franco R., Portella G., Chieffi S., Chieffi P. (2015). High levels of GPR30 protein in human testicular carcinoma in situ and seminomas correlate with low levels of estrogen receptor-beta and indicate a switch in estrogen responsiveness. J. Cell. Physiol..

[263] Jouinot A., Bertherat J. (2018). Management of endocrine disease: Adrenocortical carcinoma: Differentiating the good from the poor prognosis tumors. Eur. J. Endocrinol..

[264] Barlaskar F.M., Hammer G.D. (2007). The molecular genetics of adrenocortical carcinoma. Rev. Endocr. Metab. Disord..

[265] Zheng S., Cherniack A.D., Dewal N., Moffitt R.A., Danilova L., Murray B.A., Lerario A.M., Else T., Knijnenburg T.A., Ciriello G. (2016). Comprehensive Pan-Genomic Characterization of Adrenocortical Carcinoma. Cancer Cell.

[266] Vatrano S., Volante M., Duregon E., Giorcelli J., Izzo S., Rapa I., Votta A., Germano A., Scagliotti G., Berruti A. (2018). Detailed genomic characterization identifies high heterogeneity and histotype-specific genomic profiles in adrenocortical carcinomas. Mod. Pathol..

[267] Kiesewetter B., Riss P., Scheuba C., Mazal P., Kretschmer-Chott E., Haug A., Raderer M. (2021). Management of adrenocortical carcinoma: Are we making progress?. Ther. Adv. Med. Oncol..

[268] Alesina P.F., Walz M.K. (2020). Adrenal Tumors: Are Gender Aspects Relevant?. Visc. Med..

[269] Hsing A.W., Nam J.M., Co Chien H.T., McLaughlin J.K., Fraumeni J.F. (1996). Risk factors for adrenal cancer: An exploratory study. Int. J. Cancer.

[270] de Cremoux P., Rosenberg D., Goussard J., Bremont-Weil C., Tissier F., Tran-Perennou C., Groussin L., Bertagna X., Bertherat J., Raffin-Sanson M.L. (2008). Expression of progesterone and estradiol receptors in normal adrenal cortex, adrenocortical tumors, and primary pigmented nodular adrenocortical disease. Endocr. Relat. Cancer.

[271] Albrecht E.D., Babischkin J.S., Davies W.A., Leavitt M.G., Pepe G.J. (1999). Identification and developmental expression of the estrogen receptor alpha and beta in the baboon fetal adrenal gland. Endocrinology.

[272] Takeyama J., Suzuki T., Inoue S., Kaneko C., Nagura H., Harada N., Sasano H. (2001). Expression and cellular localization of estrogen receptors alpha and beta in the human fetus. J. Clin. Endocr. Metab..

[273] Baquedano M.S., Saraco N., Berensztein E., Pepe C., Bianchini M., Levy E., Goni J., Rivarola M.A., Belgorosky A. (2007). Identification and developmental changes of aromatase and estrogen receptor expression in prepubertal and pubertal human adrenal tissues. J. Clin. Endocr. Metab..

[274] Barzon L., Masi G., Pacenti M., Trevisan M., Fallo F., Remo A., Martignoni G., Montanaro D., Pezzi V., Palu G. (2008). Expression of aromatase and estrogen receptors in human adrenocortical tumors. Virchows Arch..

[275] Sirianni R., Zolea F., Chimento A., Ruggiero C., Cerquetti L., Fallo F., Pilon C., Arnaldi G., Carpinelli G., Stigliano A. (2012). Targeting estrogen receptor-alpha reduces adrenocortical cancer (ACC) cell growth in vitro and in vivo: Potential therapeutic role of selective estrogen receptor modulators (SERMs) for ACC treatment. J. Clin. Endocr. Metab..

[276] Montanaro D., Maggiolini M., Recchia A.G., Sirianni R., Aquila S., Barzon L., Fallo F., Ando S., Pezzi V. (2005). Antiestrogens upregulate estrogen receptor beta expression and inhibit adrenocortical H295R cell proliferation. J. Mol. Endocrinol..

[277] Vivacqua A., Bonofiglio D., Recchia A.G., Musti A.M., Picard D., Ando S., Maggiolini M. (2006). The G protein-coupled receptor GPR30 mediates the proliferative effects induced by 17beta-estradiol and hydroxytamoxifen in endometrial cancer cells. Mol. Endocrinol..

[278] Chen J.R., Plotkin L.I., Aguirre J.I., Han L., Jilka R.L., Kousteni S., Bellido T., Manolagas S.C. (2005). Transient versus sustained phosphorylation and nuclear accumulation of ERKs underlie anti-versus pro-apoptotic effects of estrogens. J. Biol. Chem..

[279] Brown J.W., Prieto L.M., Perez-Stable C., Montoya M., Cappell S., Fishman L.M. (2008). Estrogen and progesterone lower cyclin B1 AND D1 expression, block cell cycle in G2/M, and trigger apoptosis in human adrenal carcinoma cell cultures. Horm. Metab. Res..

[280] Prieto L.M., Brown J.W., Perez-Stable C., Fishman L.M. (2008). High dose 17 beta-estradiol and the alpha-estrogen agonist PPT trigger apoptosis in human adrenal carcinoma cells but the beta-estrogen agonist DPN does not. Horm. Metab. Res..

[281] Wang T., Rainey W.E. (2012). Human adrenocortical carcinoma cell lines. Mol. Cell. Endocrinol..

